# Exploring the *Saccharomyces cerevisiae* Volatile Metabolome: Indigenous versus Commercial Strains

**DOI:** 10.1371/journal.pone.0143641

**Published:** 2015-11-24

**Authors:** Zélia Alves, André Melo, Ana Raquel Figueiredo, Manuel A. Coimbra, Ana C. Gomes, Sílvia M. Rocha

**Affiliations:** 1 Departament of Chemistry & QOPNA, University of Aveiro, 3810–193, Aveiro, Portugal; 2 Genomics Unit, Biocant–Biotechnology Innovation Center, Parque Tecnológico de Cantanhede, Núcleo 4, Lote 8, 3060–197, Cantanhede, Portugal; 3 Departament of Biology & CESAM, University of Aveiro, 3810–193, Aveiro, Portugal; Colorado State University, UNITED STATES

## Abstract

Winemaking is a highly industrialized process and a number of commercial *Saccharomyces cerevisiae* strains are used around the world, neglecting the diversity of native yeast strains that are responsible for the production of wines peculiar flavours. The aim of this study was to in-depth establish the *S*. *cerevisiae* volatile metabolome and to assess inter-strains variability. To fulfill this objective, two indigenous strains (BT2652 and BT2453 isolated from spontaneous fermentation of grapes collected in Bairrada Appellation, Portugal) and two commercial strains (CSc1 and CSc2) *S*. *cerevisiae* were analysed using a methodology based on advanced multidimensional gas chromatography (HS-SPME/GC×GC-ToFMS) tandem with multivariate analysis. A total of 257 volatile metabolites were identified, distributed over the chemical families of acetals, acids, alcohols, aldehydes, ketones, terpenic compounds, esters, ethers, furan-type compounds, hydrocarbons, pyrans, pyrazines and S-compounds. Some of these families are related with metabolic pathways of amino acid, carbohydrate and fatty acid metabolism as well as mono and sesquiterpenic biosynthesis. Principal Component Analysis (PCA) was used with a dataset comprising all variables (257 volatile components), and a distinction was observed between commercial and indigenous strains, which suggests inter-strains variability. In a second step, a subset containing esters and terpenic compounds (C_10_ and C_15_), metabolites of particular relevance to wine aroma, was also analysed using PCA. The terpenic and ester profiles express the strains variability and their potential contribution to the wine aromas, specially the BT2453, which produced the higher terpenic content. This research contributes to understand the metabolic diversity of indigenous wine microflora versus commercial strains and achieved knowledge that may be further exploited to produce wines with peculiar aroma properties.

## Introduction

Winemaking is a highly industrialized process and different *Saccharomyces cerevisiae* starter cultures are commercially available for its control and fermentation homogeneity. In fact, their dominant growth reduce the development of indigenous species potentially detrimental and, as a consequence, decrease the risk of wine spoilage [1–3]. However, the current existing commercial yeasts present some constrains, namely reducing the uniqueness of wine bouquet, characteristic of the different vitivinicultural regions throughout the world [1,4,5]. Recently, the role of indigenous yeast strains has gaining importance, as a form to express peculiar characters and to leverage the aroma profile of wines from a specific region/Appellation [6–9]. Indeed, the use of a “microarea-specific” starter culture has shown that the volatile profile of wine is strictly related to the geographical origin of the yeast employed used on the fermentation process [9]. Also, the high biodiversity of *S*. *cerevisiae* strains throughout the vitivinicultural regions suggests the occurrence of specific natural strains associated with particular *terroirs* [10], which is not yet completely understood [11–13], namely those related with volatile metabolomic profiles. Previous studies suggested that some yeasts volatile metabolites, such as alcohols, esters and organic acids have impact on wine character [2,11].

Microbial metabolomics constitutes an integrated component of systems biology, as it studies a set of metabolites produced by microorganism and monitors the global outcome of interactions between their development processes and the environment. Thus, metabolomics can potentially provide a more accurate snapshot of the physiological state of the microorganisms [14]. Recently, a metabolomic research exposed different behaviours of *S*. *cerevisiae* in two distinct growth media, expressed by differences in exometabolites related to wine quality, especially on the higher alcohols [15]. Studies on the characterization of *S*. *cerevisiae* metabolome by using high resolution ^1^H NMR (Nuclear Magnetic Resonance) [16] and GC-MS (gas chromatography-mass spectrometry) [17] were also reported, providing evidences that yeast behaviour depends on the strain, namely on the response to the stress induced by ethanol [16], and on the production of volatile aroma compounds [17]. Furthermore, different nutrients availability (e.g. nitrogen supplementation) confers strain metabolic behaviour changes during fermentative process, such variability is not only determined as a result of the stress but also due to the genetic background of the strain [18]. Actually, microbial metabolomics has been a breaking new ground as a very useful tool in several areas, including those related to microbial technology and aroma properties, since microorganisms produce several volatile metabolites that can be used as unique chemical fingerprints of each species, and possibly of each strain. Thus, the aim of the current research was to in-depth establish the *S*. *cerevisiae* metabolome based on the volatile metabolites. To fulfil this objective, two indigenous strains (isolated from spontaneous fermentation of grapes collected in Bairrada Appellation, Portugal) and two of the mostly used commercial strains (CSc1 and CSc2) of *S*. *cerevisiae* were analysed using high sensitive and high throughput methodology based on two dimensional gas chromatography (GC×GC). Previously, a phenotypic characterization was performed to allow a selection of the two *S*. *cerevisiae* indigenous strains using some crucial criteria such as high resistance to copper sulphate, high temperature tolerance, low production of hydrogen sulphide, killer activity, high tolerance to sulphur dioxide and high fermentation performance, that unveil the yeast oenological potential [19].

## Materials and Methods

**Fig 1:**
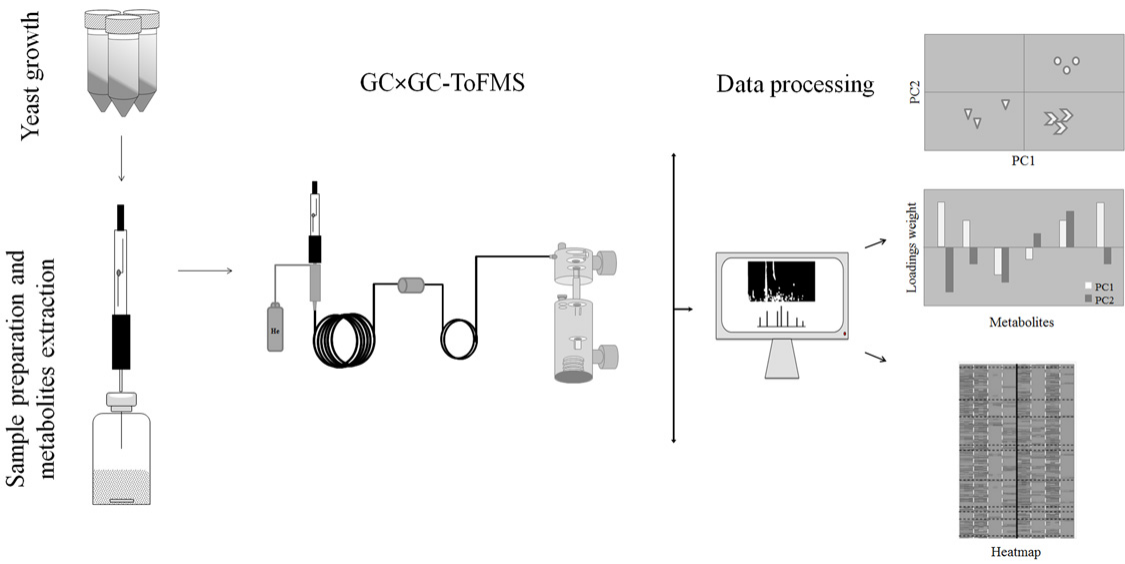
Schematic representation of the main stages for *S*. *cerevisiae* metabolome determination. This includes yeast growth, sample, preparation and metabolites extraction, GC×GC analysis and data processing.

The sampling, reporting of chemical analysis and metadata relative to data pre-processing, pre-treatment, processing, validation and interpretation were performed according to the metabolomics standards initiative (MSI) [20–22]. Fig 1 represents the main stages for *S*. *cerevisiae* metabolome determination, including yeast growth, sample preparation and metabolites extraction, GC×GC analysis and data processing, which were described in detail in the following sub-sections.

### *S*. *cerevisiae* isolation

Grape samples were collected in Bairrada Appellation (Portugal) in the 2011 vintage. In the laboratory, grapes were crushed in a bag in aseptic conditions and the resulting must was placed in a sterile 500 mL Erlenmeyer. The fermentations were carried out at room temperature. The fermentation kinetic was monitored by daily weight measurements until the must weight was reduced 70 g/L (which was close to the end of fermentation). At the end of the fermentation, samples were filtered, diluted 10^−4^ times and plated in agar YPD medium (1% w/v yeast extract, 2% w/v glucose, 2% w/v peptone) in an incubator at 30°C during 48 h. Then, 30 randomly chosen colonies were collected and the isolates were cryopreserved in glycerol (40%, w/v) at—80°C.

The molecular identification of the isolated yeasts was carried out as described in [23] and in S1 Protocol and S1 Fig, both from Supporting Information. A total of 313 *S*. *cerevisiae* strains were identified. In order to select a reduced number of yeast strains to carry out the HS-SPME/GC×GC-ToFMS analysis, the phenotypic traits of the entire set of strains were characterized, to expose the maximum variability within the isolated strain collection. Thus, it was used a high throughput phenomics methodologies (S2 Protocol from Supporting Information), where a total of 36 growth conditions were tested, and grouped according to their phenotype profile. For the selection of two *S*. *cerevisiae* strains to use in downstream experiments, the following criteria have taken into consideration: firstly, strains were trimmed according to their oenological potential, where strains with high tolerance to SO_2_ and CuSO_4_, that were able to grow at high temperatures (42°C), and did not produce H_2_S were selected; secondly, from the resulting reduced set of strains, those two that displayed a more distinctive phenotype profile were selected (BT2453 and BT2652) (S2 Fig from Supporting Information). Also, two of the mostly used commercial wine yeast strains were selected for the volatile metabolome analyses (herein referred as CSc1 and CSc2).

### Growth conditions for metabolomics characterization

The cryopreserved yeasts (BT2453, BT2652, CSc1 and CSc2) were plated in YPD (yeast peptone dextrose, Formedium, Norfolk, UK) solid medium and incubated during two days at 30°C. Then, an isolated colony was replied from the medium and transferred into a falcon tube (50 mL) containing 10 mL of YPD liquid medium to grow at 30°C overnight, with gentle stirring (100 rpm). Afterwards, the cells were counted with a TC10 Automated Cell Counter (BioRad) and 10^6^ cells of each strain were transferred to 20 mL of SD (0.69% (w/v) from YNB (yeast nitrogen base) without amino acids and 2% (w/v) from glucose, Formedium, Norfolk, UK) liquid medium and incubated at 30°C during 25 h, with a stirring of 160 rpm. The SD medium was chosen as a minimal growth media to allow a reduce co-elution with the yeast volatile components and metabolites. Three independent assays were performed for each strain. The cell culture was transferred to a vial of 60 mL with a magnetic stirrer and 4 g of NaCl 99.5%. The vial was capped with a PTFE septum and an aluminium screw cap (Chromacol, Hertfordshire, UK) and the metabolic quenching was achieved by freezing the samples at -80°C. The strict control of quenching procedure that arrests the cellular metabolism and enzymatic reactions of yeast should be done to reduce data variability. As a control, 20 mL of SD medium in vials of 60 mL with a magnetic stirrer and 4 g of NaCl 99.5% was used.

### *S*. *cerevisiae* metabolome profiling by HS-SPME/GC×GC-ToFMS

The volatile metabolites of the four yeast strains were analysed by headspace solid phase microextraction (HS-SPME) combined with comprehensive two-dimensional gas chromatography coupled to mass spectrometry with a high resolution time of flight analyzer (GC×GC-ToFMS). The SPME device included a fused silica fiber coating partially cross-linked with 50/30 μm divinylbenzene/carboxen/poly(dimethylsiloxane) (DVB/CAR/PDMS), with 1cm of length. The SPME fiber was conditioned at 250°C for 30 min in the GC injector, according to the manufacturer’s recommendations.

For the HS-SPME assay, the samples stored at– 80°C in sampling vials were dethawed and placed in a thermostated water bath adjusted to 40.0 ± 0.1°C for 15 min to promote the transference of the metabolites from the sample to the headspace. After this step, the SPME fiber coating was manually inserted into the sample vial headspace for 45 min to obtain the free volatile metabolites. Three independent cultures were analysed from each *S*. *cerevisiae* strain.

After the extraction/concentration step, the SPME fiber was manually introduced into the GC×GC-ToFMS injection port at 250°C and maintained for 30 s for desorption. The injection port was lined with a 0.75 mm I.D. splitless glass liner. Splitless injections were used (30 s). The LECO Pegasus 4D (LECO, St. Joseph, MI, USA) GC×GC-ToFMS system consisted of an Agilent GC 7890A gas chromatograph (Agilent Technologies, Inc., Wilmington, DE), with a dual stage jet cryogenic modulator (licensed from Zoex) and a secondary oven, and a mass spectrometer equipped with a high resolution ToF analyser. An Equity-5 column (30 m × 0.32 mm I.D, 0.25 μm film thickness, J&W Scientific Inc, Folsom, CA, USA) was used as ^1^D (first dimension) column and a DBFFAP (0.79 m × 0.25 mm I.D., 0.25 μm film thickness, J&W Scientific Inc, Folsom, CA, USA) was used as a ^2^D (second dimension) column. The carrier gas was helium at a constant flow rate of 2.50 mL/min. The primary oven temperature was programmed from 40°C (hold 1 min) to 230°C (10°C/min) (hold 2 min). The secondary oven temperature program was 30°C offset above the primary oven. The MS transfer line and MS source temperature was set at 250°C. The modulation time was 5 s and the modulator temperature was kept at 20°C offset (above secondary oven). The ToFMS was operated at a spectrum storage rate of 125 spectra/s. The mass spectrometer was operated in the EI mode at 70 eV using a range of *m/z* 35–350 and the detector voltage was -1786 V. Total ion chromatograms (TIC) were processed using the automated data processing software ChromaTOF^®^ (LECO) at signal-to-noise threshold of 100. Contour plots were used to evaluate the separation general quality and for manual peak identification. In order to tentatively identify the different compounds, the mass spectrum of each compound detected was compared to those in mass spectral libraries which included an in-house library of standards, and commercial databases (Wiley 275 and US National Institute of Science and Technology (NIST) V. 2.0 -Mainlib and Replib). Moreover, a manual analysis of mass spectra was done, combining additional information like retention index (RI) value, which was experimentally determined according to van den Dool and Kratz RI equation [24]. A C_8_-C_20_ *n*-alkanes series was used for RI determination (the solvent *n*-hexane was used as C_6_ standard), comparing these values with reported ones in existing literature for chromatographic columns similar to ^1^D column above mentioned (S1 Table from Supporting Information). The majority of the identified compounds presented similarity matches > 800. The Deconvoluted Total Ion Current (DTIC) GC×GC area data were used as an approach to estimate the relative content of each metabolite component of yeast strains. Reproducibility was expressed as relative standard deviation (% RSD) in S1 and S2 Tables from Supporting Information.

### Data processing

In order to extract the main sources of variability and hence to characterize the data set, a multivariate analysis was performed. Principal Component Analysis (PCA) is a statistical tool used to visualize patterns of classification from the data sets analysed. It simplifies high dimensionality data sets by converting observations into principal components that emphasise variances in the data [25]. Peak areas of all compounds were extracted from the chromatograms and used to build the data matrix, consisting of 12 observations (3 independent samples per strains, in a total of 4 yeast strains) and 257 variables (metabolites). A complete list of these compounds is provided in S1 Table and the full data set with the corresponding areas of the three independent replicates of each strain is presented in S2 Table (Supporting Information). Considering the relevant potential contribution of the terpenic compounds and esters for the wine aroma, in a second step, a PCA was also performed using these two chemical families. This sub-data set consisted of 12 observations (3 independent samples per strain, in a total of 4 yeast strains) and 72 variables (peak areas of terpenic compounds and esters). After median and autoscaling normalization, PCAs were performed using the MetaboAnalyst 2.0 (web interfaces) [26,27]. A heatmap visualization of the data set (4 yeast strains and 257 volatile compounds) was also performed after a logarithm function transformation of each GC peak area using the Unscrambler® X (30 day trial version–CAMO Software AS, Oslo, Norway).

## Results and Discussion

### *S*. *cerevisiae* metabolome profiling

**Fig 2:**
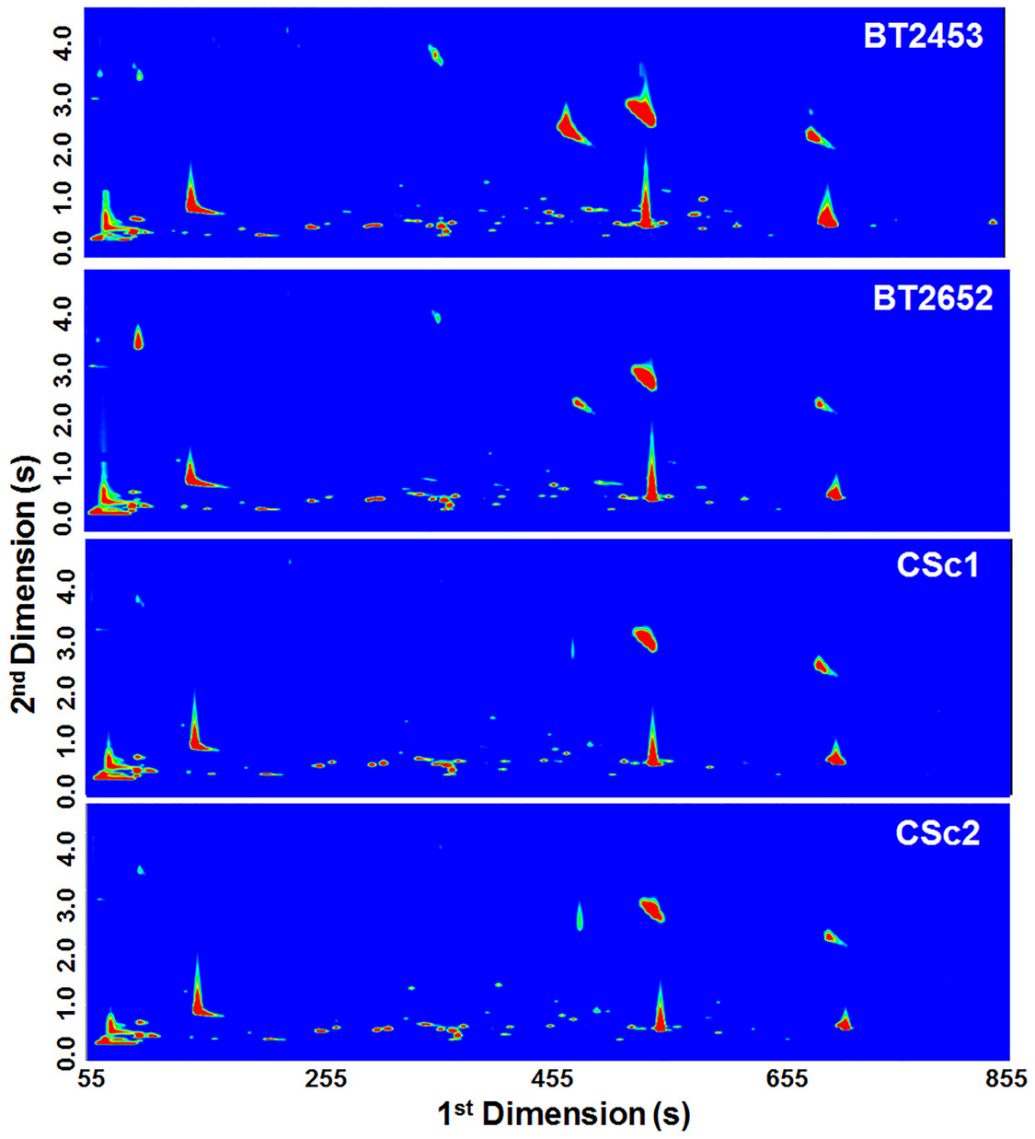
Volatile metabolome of *S*. *cerevisiae* strains represented by GC×GC total ion chromatogram contour plots: BT2453 and BT2652 corresponding to strains isolated from wine, and CSc1 and CSc2, corresponding to commercial yeast strains.

The volatile metabolome of four *S*. *cerevisiae* strains under study is represented in the GC×GC total ion chromatogram contour plots (Fig 2). The visual analysis of the contour plots allows a rapid assessment of yeast strains comparison. This technique is ideal for the analysis of complex mixtures, as compounds with similar chemical structure can be grouped into distinct patterns in the 2D chromatographic plane, providing useful information on their volatility and polarity (as a non-polar/polar column set was used), and relationships of structured retentions have proved especially useful for compound identification (S1 Table). This unique peculiarity of the GC×GC chromatogram is a powerful tool in the identification step.

The contour plot shows that all *S*. *cerevisiae* strains have similar profiles (Fig 2), although minimal differences between yeast strains are noticeable. In fact, BT2453 strain exhibited higher number of compounds and peak areas. For a detailed *S*. *cerevisiae* metabolomic profiling, a pre-processing of raw instrumental data was done to construct a data matrix regarding further statistical analysis. The full data matrix is provided as Supplementary Data (S1 Table), which presents the information obtained for each of the 257 compounds tentatively identified, including the retention times in both dimensions, GC peak areas, RSD, and retention index experimentally calculated (RI_cal_) and available in the literature for a 5% phenyl polysilphenylene-siloxane GC column or equivalent (RI_lit_).

**Fig 3:**
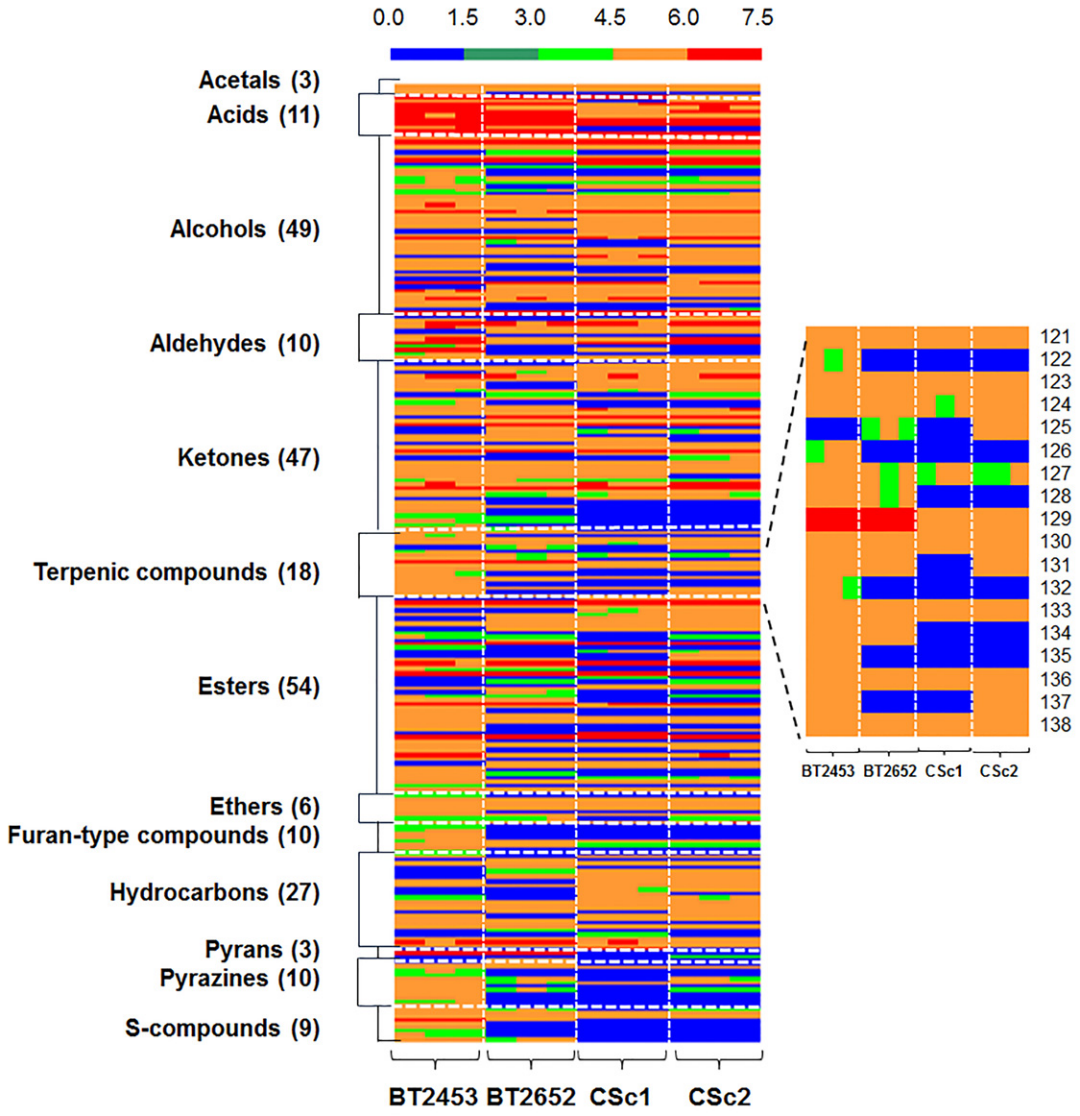
Heatmap representation of GC×GC peak areas of metabolites from four *S*. *cerevisiae* strains. The metabolites were organized by chemical families, and with the indication of the number of compounds per family. Areas are normalized by applying a logarithm function. Each line corresponds to one metabolite, and each column corresponds to each strain (BT2453, BT2652, CSc1 and CSc2, each one with three independent cultures). The terpenic profile is highlighted at the right side: β -Myrcene (**121**), α-Terpinene (**122**), Limonene (**123**), β-Ocimene (**124**), Dihydromyrcenol (**125**), γ-Terpinene (**126**), 6,10-Dihydromyrcenol (**127**), α-Terpinolene (**128**), Linalool (**129**), α-Terpineol (**130**), Nerol (**131**), Isogeraniol (**132**), Geraniol (**133**), Citral (**134**), Geranylacetate (**135**), β-Farnesene (**136**), α-Farnesene (**137**), Nerolidol (**138**).

The volatile metabolites were distributed over 13 chemical classes including acetals, acids, alcohols, aldehydes, ketones, terpenic compounds, esters, ethers, furan-type compounds, hydrocarbons, pyrans, pyrazines and S-compounds (Fig 3 and S1 Table), revealing the complexity of this matrix. To date, studies on the volatile metabolome of *S*. *cerevisiae* have reported the presence of 1,1-diethoxyethane, 1-propanol, isobutanol, 3-methylbutanol, 2-methylbutanol, 2-phenylethanol, acetoin (3-hydroxy-2-butanone), ethyl acetate, ethyl lactate (ethyl 2-hydroxypropanoate) previously reported [15]. Further, other studies performed on wine characterization also suggests the role of the yeasts on the production of aroma compounds, such as acetic acid, hexanoic acid, octanoic acid, ethyl hexanoate, ethyl octanoate, 2-phenylethyl acetate, butanal, 3-methyl-1-butanal, 1-hexanol, linalool, geraniol or nerol [2,28,29]. These metabolites were also detected on the present research as *S*. *cerevisiae* metabolites.

The number of metabolites per strain ranged from 210 compounds for BT2453 strain up to 156 for the CSc1 strain. The CSc2 and the BT2652 strains produced similar amounts of volatile compounds, 172 and 170, respectively. For an easy interpretation of the full data set concerning the volatile profile of the strains under study a heatmap representation (Fig 3) was performed, which allows a rapid visual assessment of the similarities and differences between samples. Concerning the number of compounds identified, esters, alcohols and ketones represent the major groups for all the *S*. *cerevisiae* strains (Fig 3). Indeed, differences on the relative abundance of compounds among strains were observed, suggesting that these strains have different metabolome profiles. The BT2453 strain, which stands out from the other three, was isolated from wine and produced the higher relative abundances of compounds belonging to the chemical family of aldehydes, terpenic compounds, esters, furan-type compounds, pyrazines and S-compounds.

The *S*. *cerevisiae* metabolome profiling revealed a network of pathways that explains the origin of the detected metabolites. This information is scarce and disperse, however, the origin of some families is already known. For instance, aliphatic acids with a short chain, such as acetic, propanoic and butanoic acids, are by-products of fermentation, while medium-chain fatty acids (e.g. octanoic and decanoic acids) are intermediates formed enzymatically during fermentation by fatty acid biosynthesis [2,30]. The octanoic acid was the most dominant acid for all the strains under study, followed by decanoic acid, excepting for BT2652 strain, whose second most abundant compound was acetic acid. The biosynthesis of higher alcohols derives either by Ehrlich pathway (transamination reactions of an amino acid to an α-keto acid) and/or by carbohydrate metabolism via pyruvate by anabolic pathway. The resulting *α*-keto acid is converted into the corresponding fusel alcohol by decarboxylation to form an aldehyde and followed by a reduction to higher alcohols [2,31]. The 3-methyl-1-butanol and 2-methyl-1-propanol were the main aliphatic alcohols present in the metabolome from all yeast strains, and both can be produced through degradation of the branched-chain amino acids leucine and valine. Other relevant volatile metabolites are 2-methyl-1-butanol and 2-phenylethanol, which can be formed from isoleucine and phenylalanine, respectively [2,31]. Both aliphatic (eg. 3-methyl-1-butanal and 2-methyl-1-butanal) and aromatic aldehydes (e.g. 2-phenylacetaldeyde) can derive from degradation of specific amino acids and from sugar metabolism. Further, this chemical family can be formed by oxidation of alcohols and autoxidation of fatty acids [2]. Some aliphatic ketone metabolites are produced by *S*. *cerevisiae* strain as intermediates in the reductive decarboxylation of pyruvate to 2,3-butanodiol (acetoin biosynthesis), generally 2,3-butanedione (diacetyl) and 3-hydroxy-2-butanone (acetoin). These compounds can be produced during amino acid, especially valine, and sugar metabolism [32]. Sulphur compounds family is characterized by some chemical classes as ketones, alcohols, furans and thiazoles. The degradation of sulphur-containing amino acids (methionine and cysteine) by Ehrlich pathway is responsible of volatile sulphur compounds (e.g. 3-(methylthio)propanol and methyldisulfide) [31,33].

**Fig 4:**
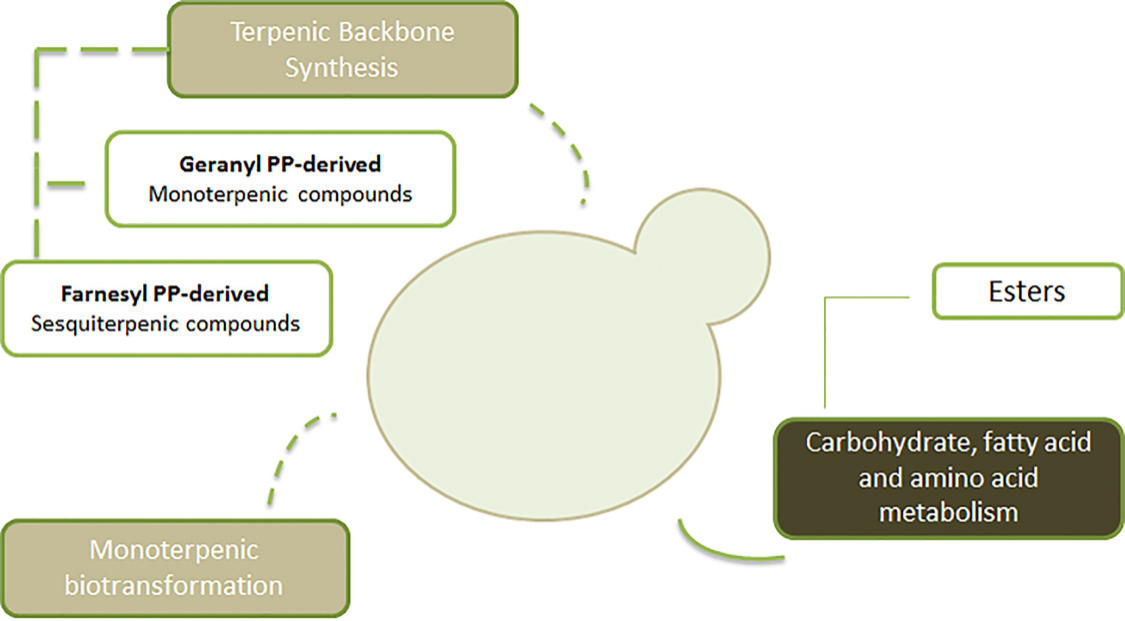
Schematic representation proposed to explain *S*. *cerevisiae* metabolic pathways related to terpenic and esters chemical families. (PP—Diphosphate) [34,35,37].

From all the 13 chemical families identified on the *S*. *cerevisiae* metabolome, particular attention was done to the terpenic and esters compounds due to their potential positive impact to the wine aroma properties. Regarding terpenic compounds, *S*. *cerevisiae* metabolome comprised 9 up to 17 metabolites per strain, in a total of 18 compounds detected on all the strains under study, distributed over monoterpenic (15) and sesquiterpenic (3) compounds. Hydrocarbon-type compounds represent the higher number of components, however the higher content was observed for the alcohol type, such as for the linalool, geraniol and nerolidol. The BT2453 strain presented the higher total GC peak area due to the high abundance of linalool and the presence of four metabolites which were not present in volatile metabolome of other strains (α-terpinene, γ-terpinene, iso-geraniol and nerol acetate), thus explaining its potential contribution for peculiar beverage characteristics. The origin of mono and sesquiterpenic compounds may be explained based on a set of mechanisms: terpen biosynthesis pathway (mevalonate pathway where the geranyl and farnesyl diphosphate, precursors of monoterpenic and sesquiterpenic compounds respectively, are produced) [5,34] and/or biotransformation reactions [35,36] from precursor compounds present in growth media composition (four monoterpenoids, γ-terpinene, α-terpinolene, linalool, α-terpineol) was found in SD growth medium–data not shown). A schematic representation is proposed to illustrate the network of pathways that explains the *S*. *cerevisiae* terpen and ester metabolites origin (Fig 4).

Esters family is characterized by 45 aliphatic (C_4_-C_15_) and 9 aromatic (C_8_-C_12_) compounds, which are represented by acetates of higher alcohols and ethyl esters of fatty acids [38]. The first can be formed by condensation reaction between acetyl-CoA and alcohol [38] and the second is derived from ethanol and a medium-chain acyl-CoA activated [2,29,39]. These substrates are produced during carbohydrate, fatty acid and/or amino acid metabolism [37]. Ethyl octanoate represented the major ester for all the strains, with the exception of CSc1. For this strain, ethyl decanoate was the dominant one. Other relevant compound that characterizes the volatile metabolome for all strains was ethyl hexanoate, and the BT2652 strain was the higher producer. Ethyl dec-9-enoate was only detected in the metabolome profile of CSc1. Within aromatic esters, BT2453 strain produced all the metabolites while the remaining strains only produced four. However, all the strains were capable to produce the two major aromatic esters tentatively identified in *S*. *cerevisiae* volatile metabolome—methyl benzoate and 2-phenylacetate. In relation to aliphatic acetates, ethyl acetate and 2-methylbutyl acetate were the major components for all strains under study. Ethyl acetate can be one of the major compounds due to the large quantities of ethanol present and because the primary alcohols are the most reactive [40].

### *S*. *cerevisiae* strains distinction using Principal Component Analysis and potential impact on aroma peculiarities

**Fig 5:**
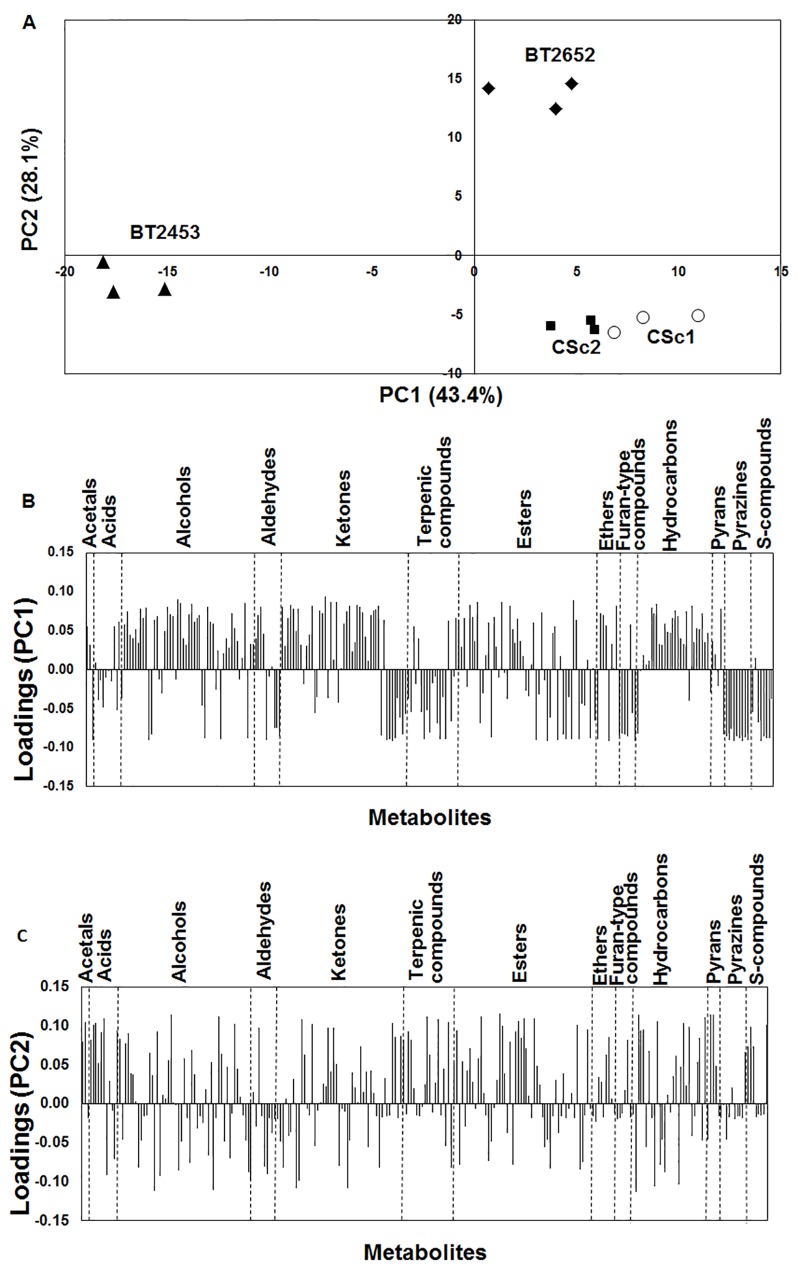
PC1 × PC2 analysis applied to all chemical families from metabolome of the four yeasts strains: BT2453, BT2652, CSc1 and CSc2. Scores scatter plot (A) and PC1 (B) and PC2 (C) loadings plots.

In order to simplify the high dimensionality of the data set and to understand the variability among the strains, the GC peak areas of 257 metabolites were submitted to a PCA procedure to generate a visual representation of the discrimination between the strains by their metabolite profiles. Fig 5(A) shows the scores scatter plot in which the two first principal components explain 71.5% of the total variability of data set. Fig 5(B) and 5(C) represents the corresponding PC1 and PC2 loadings plot, respectively, which establishes the contribution of each metabolite to the principal components. Accordingly, PC1 explains 43.4% of the total variability, and BT2453 strain (PC1 negative) was distinguished from all other strains, and was characterized by aldehydes, ketones, terpenic compounds, esters, furan-type, pyrazines and S-compounds. PC2, which explains 28.1% of the total variability, allowed for the distinction of BT2652 strain (PC2 positive) from the two commercial strains (CSc1 and CSc2). The commercial strains were clustered, allowing to infer their similar metabolomic signature. These strains were predominantly characterized by alcohols, ketones and hydrocarbons families. On the other hand, the indigenous strain BT2652 was characterized by acetals, alcohols, ketones, esters, ethers and hydrocarbons compounds.

**Fig 6:**
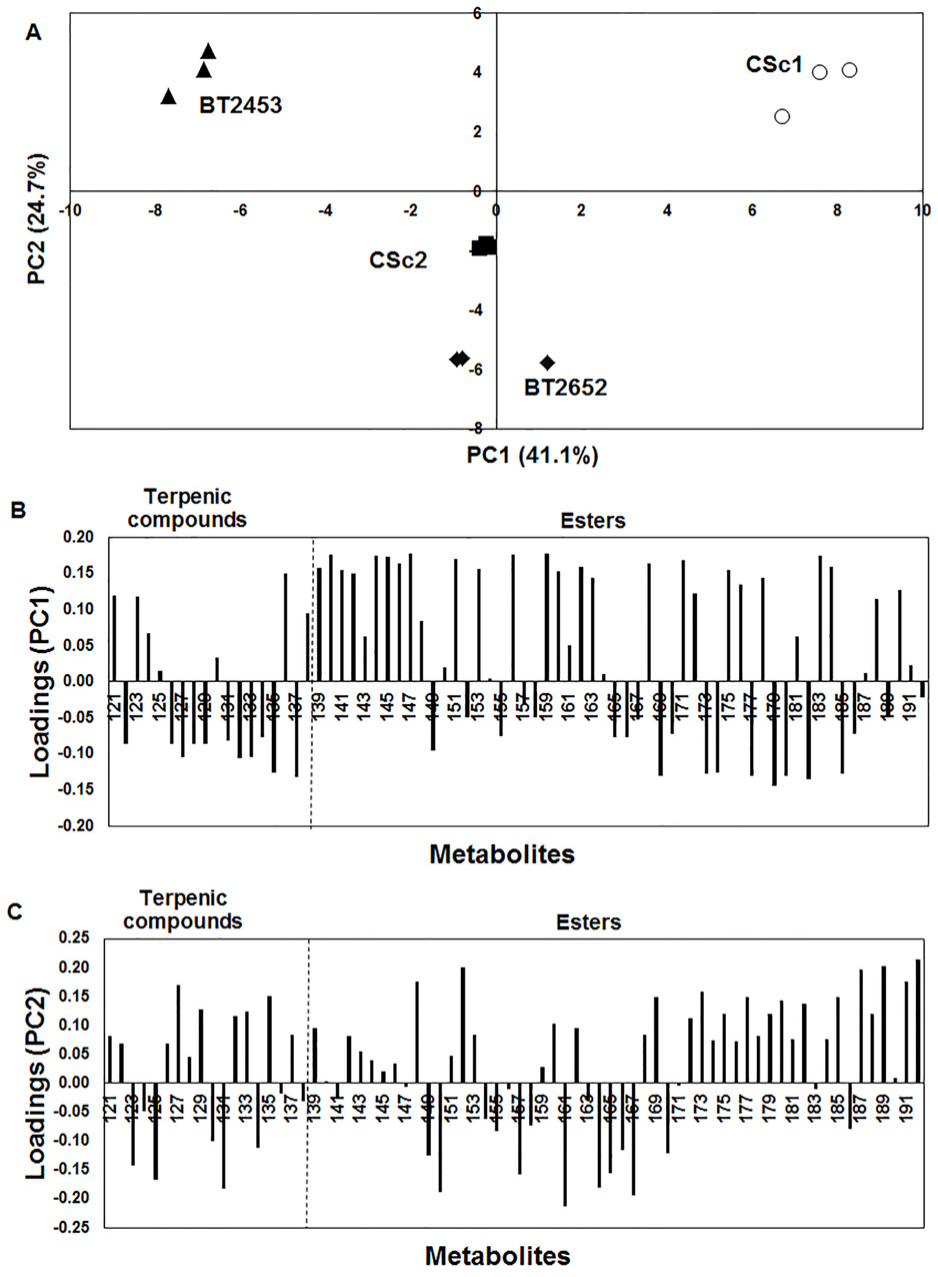
PC1 × PC2 analysis applied to terpenic compounds and esters from metabolome of the four yeasts strains: BT2453, BT2652, CSc1 and CSc2. Scores scatter plot (A) and PC1 (B) and PC2 (C) loadings plots.

Due to the complexity of the PC1 × PC2 loadings (Fig 5) and to potential relevant impact of esters and terpenic compounds on the aroma properties, a second multivariate analysis by PCA was performed using these two chemical families. Fig 6(A) represents the PC1 × PC2 scores plot, which explains 65.8% of the total variability. PC1 allowed to distinction of three clusters, where the commercial CSc1 and BT2453 indigenous strain were projected in PC1 positive and negative, respectively, and the other commercial strain CSc2 and BT2652 indigenous strain were near to origin. PC2 allowed the distinction between BT2652 indigenous wine strain and CSc2 commercial strain.

Fig 6B and 6C represents the corresponding PC1 and PC2 loadings plot, respectively, which establishes the contribution of each metabolite to the principal components. To estimate the potential impact of each strain on aroma properties, the loadings strain attribution was done in parallel with the aroma descriptor of each variable (metabolite). BT2453 is characterized by 9 of 18 terpenic compounds present in volatile metabolome profile such as α-terpinene (herbal, citrus), γ-terpinene (citrus, menthol), 6,10-dihydromyrcenol, α-terpinolene (sweet pine, lime, green), linalool (citrus, sweet, floral) [41,42], isogeraniol (floral), geraniol (floral, sweet) [41], geranyl acetate (fresh, rose) and α-farnesene (woody), and aliphatic long chain and aromatic esters—butoxyethanol acetate, decyl acetate (fruity), propyl decanoate, 2-methylpropyl decanoate, dodecyl acetate, benzyl acetate (floral, bitter), 4-ethylphenyl acetate and ethyl 3-phenylpropanoate. CSc1 is characterized by esters with short chain like propyl formate, ethyl acetate (vinegar, fruity), propyl acetate (fruity), ethyl isobutyrate (fruity, strawberry), isobutyl acetate (floral), isopentyl formate (sweet, green, fruity, winey), ethyl butyrate (fruity) and ethyl 2-hydroxypropanoate (buttery). CSc2 and BT2652 exhibited a similar terpenic composition: limonene (citrus) [43], β-ocimene (green) [44], dihydromyrcenol, α-terpineol (floral, sweet) [41], nerol (rose) [41], citral (sweet, lemon) [43] and nerolidol (floral) [44]–and esters with medium chain–ethyl 2-butenoate (caramellic fruity), pentyl propanoate (sweet, fruity, apricot-pineapple), 3-acetyloxypropyl acetate, butyl butanoate, isobutyl hexanoate, ethyl 7-octenoate, ethyl octanoate (fruity, floral).

These results highlight the metabolomic inter-strains variability and, consequently, their different impact on wine aroma. BT2453 may confer a uniqueness character peculiar due to its ability to produce terpenes. Conversely, CSc1 strain, which is currently used in winemaking, was mainly distinguished from the others due to its capacity to produce short chain esters. Finally, it is important to point out that the effective impact of these strains on the aroma properties will depended on a network of effects, such as strain metabolism, raw material composition and winemaking procedure, amongst others.

## Concluding Remarks

In this work metabolic variability of *S*. *cerevisiae* strains were analyzed in a minimal growth medium. The in-depth metabolome characterization by GC×GC-ToFMS combined with SPME revealed the complexity of the matrix under study, and a high number of instrumental features were detected from the 257 metabolites identified. It was possible to highlight that their origin results from a network of pathways, namely from amino acid, fatty acid and carbohydrate metabolism, as well mono and sesquiterpenoid biosynthesis. The metabolome profile of the two indigenous (isolated from spontaneous fermentation of grapes collected in Bairrada Appellation, Portugal) and the two commercial (CSc1 and CSc2) *S*. *cerevisiae* strains revealed inter-strains metabolic variability. A sub-set of metabolites belonging to terpenic and esters chemical families also showed variability between stains, suggesting that according to their metabolome profiling, these strains might produce wines with different aroma properties, namely those associated to specific fruity and floral notes. Finally, in order to go further in the understanding of the *S*. *cerevisiae* volatile metabolites, a more extensive study should be conducted using yeasts collected in different Appellations. Also, further studies are required in particular to better understand the relationship between the volatile metabolome of strains and the correspondent wine aroma profile produced. This knowledge should be crucial to produce wines with peculiar and differentiate characteristics, highlighting regional specificities which are in line with the actual market trend.

## Supporting Information

S1 Fig Workflow summarizing the *S*. *cerevisiae* selection for metabolome study. (TIFF)

S2 Fig Phenotypic characterization of the *S*. *cerevisiae* strains, with the localization on the heatmap of the 2 selected strains–BT2652 and BT2453, ordered by hierarchical clustering (Pearson Correlation with average linkage) using MeV 4.9.0. Heatmap rows = 36 growth conditions, columns = 313 endogenous *S*. *cerevisiae* strains.
(TIFF)

S1 Protocol *S*. *cerevisiae* strain isolation, identification and selection. (PDF)

S2 Protocol Phenotypic *S*. *cerevisiae* characterization. (PDF)

S1 Table Volatile metabolites identified by HS-SPME/GC×GC-ToFMS in four *S*. *cerevisiae* strains under study: BT2453 and BT2652 corresponding to strains isolated from wine, and CSc1 and CSc2, corresponding to commercial yeast strains. (PDF)

S2 Table Full data set used for statistics processing including the volatile metabolites identified by HS-SPME/GC×GC-ToFMS in four *S*. *cerevisiae* strains under study: BT2453 and BT2652 corresponding to strains isolated from wine, and CSc1 and CSc2, corresponding to commercial yeast strains. (XLS)
