# Supplementary figures and images for: Exploring the Saccharomyces cerevisiae Volatile Metabolome: Indigenous versus Commercial Strains
Source: PLoS One. 2015 Nov 24;10(11):e0143641. doi: 10.1371/journal.pone.0143641 (PMC4657929; doi:10.1371/journal.pone.0143641)

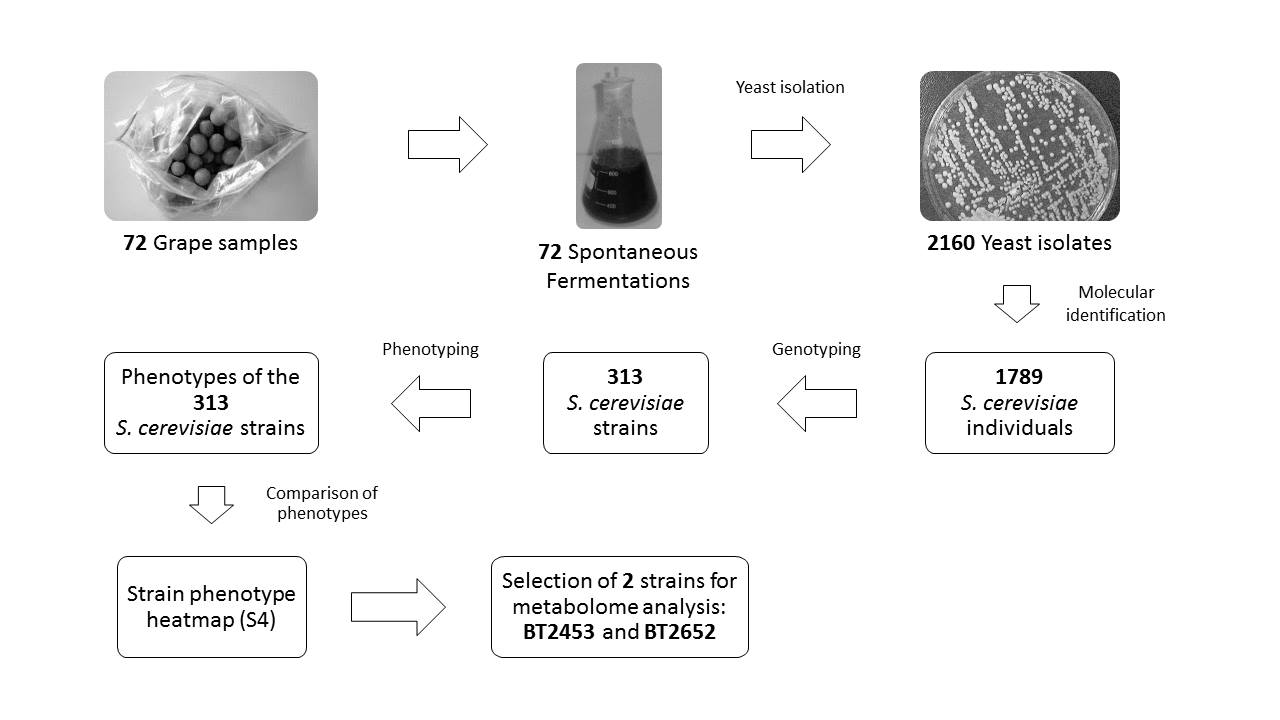

Supplement: S1 Fig — (TIFF) [file pone.0143641.s001.tiff]

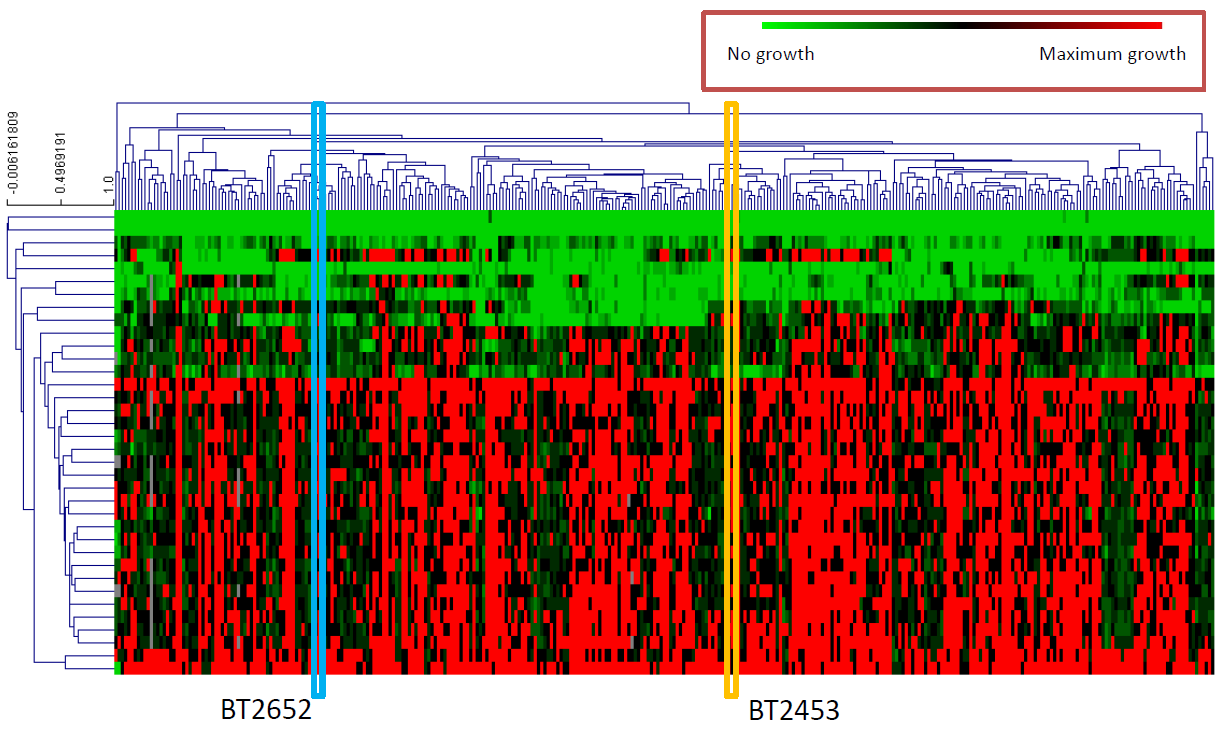

Supplement: S2 Fig — Heatmap rows = 36 growth conditions, columns = 313 endogenous S. cerevisiae strains. (TIFF) [file pone.0143641.s002.tiff]
