# Supplementary material for: Exploring the Saccharomyces cerevisiae Volatile Metabolome: Indigenous versus Commercial Strains
Source: PLoS One. 2015 Nov 24;10(11):e0143641. doi: 10.1371/journal.pone.0143641 (PMC4657929; doi:10.1371/journal.pone.0143641)
Supplement: S1 Protocol — (PDF) [file pone.0143641.s003.pdf]

Supporting Information – S1 Protocol

Exploring the *Saccharomyces cerevisiae* volatile metabolome: indigenous versus commercial strains

Zélia Alves[1], André Melo[2,3], Ana Raquel Figueiredo[1,2], Manuel A. Coimbra[1], Ana C. Gomes[2], Sílvia M. Rocha[1, *]

[1]Departament of Chemistry & QOPNA, University of Aveiro, 3810-193 Aveiro, Portugal

[2]Genomics Unit, Biocant – Biotechnology Innovation Center, Parque Tecnológico de Cantanhede, Núcleo 4, Lote 8, 3060-197 Cantanhede, Portugal

[3]Departament of Biology & CESAM, University of Aveiro, 3810-193 Aveiro, Portugal

S1 Protocol - *S. cerevisiae* strain isolation, identification and selection

Isolation of yeast strains from spontaneous fermentations

The grapes were collected in different vineyards from the Bairrada Appellation in appropriate sterile bags and sent to the laboratory in cooled boxes. In the laboratory, grapes were crushed in a bag in aseptic conditions and the resulting must was transferred to a sterile 500 mL Erlenmeyer (Duran, Mainz, Germany). All fermentations were carried out at room temperature and monitored every day by measuring the weight loss. At the end of the fermentation (70 g/L weight loss), 100 mL were collected, and filtered into falcon tubes (VWR international, Radnor, PA, USA). The filtered must was diluted to 10^{-4} times and plated in agar YPD medium (yeast extract 1% (w/v), glucose 2% (w/v), peptone 2% (w/v), agar 2%, Formedium, Norfolk, UK) and placed in an incubator (Memmert, Schwabach, Germany) at 30ºC during 48 hours. Thirty pure cultures from each fermentation were isolated and grown in the same conditions and preserved in YPD liquid medium with 40% w/v glycerol (Prolabo BDH, Fontenay-sous-Bois, France) at -80ºC. We have collected 2160 isolates from 72 fermentations.

Genomic DNA isolation

DNA isolation was done on 1 mL pure cultures, cells were collected by centrifugation and washed twice with ultrapure water. Cells were then resuspended in 100 µL of lysis buffer (1 M sorbitol, (BDH Laboratory Supplies, Poole, England) 0.1 M EDTA-Na_2 (BDH chemical, Poole, UK), 7.5 pH) and then 25 U of lyticase (Sigma-Aldrich, St. Louis, MO, USA) were added. The mixture was briefly vortexed and incubated at 37 ºC during 1 hour. Then 100 µL of a second solution (50 mM Tris-HCl, 20 mM EDTA-Na_2, 7.4 pH) and 5 µL of 10% w/v SDS (Qbiogene) were added, the mixture was vortexed and incubated at 65 ºC during 10 minutes. After that, 80 µL of 5 M potassium acetate (Sigma-Aldrich, St. Louis, MO, USA) were added and the samples were incubated on ice during at least 10 minutes. The cellular debris and proteins present were pelleted by centrifugation and the supernatant, containing the DNA was collected into a new tube with 1 volume of isopropanol (Merck, Darmstadt, Germany). The solution was mixed and incubated for 10 minutes at -20ºC allowing for DNA precipitation, which was then recovered by another centrifugation step. The supernatant

was discarded and the DNA pellet was washed with 70% ethanol (Scharlab, Barcelona, Spain) and dried for 3 minutes under vacuum. Finally, DNA was resuspended in 50 µL of TE (10mM Tris-HCl, 1 mM EDTA-Na_2, 7.5 pH) and stored at -20 ºC [1].

***S. cerevisiae* identification**

To rapidly discriminate between *Saccharomyces* and non-*Saccharomyces* yeasts, MET2 gene amplifications were performed in an Eppendorf mastercycler pro (Eppendorf, Hamburg, Germany) using synthetic oligonucleotide primers for MET2. Amplifications were carried out in 25 µL reaction volumes containing 100 ng DNA, 1x reaction buffer (100mM Tris-HCl (pH 8.8 at 25 ºC), 500 mM KCl, 0.8% igepal), 1.7 mM $MgCl_2$, 0.2 µM of each primer (MET2_F: CGA AAA CGC TCC AAG AGC TGG and MET2_R: GAC CAC GAT ATG CAC CAG GCA), 0.2 mM of each dNTP (Bioron, Ludwigshafen, Germany) and 0.5 U/µL taq DNA polymerase. After initial denaturation (94 °C for 3 minutes), the reaction mixture was cycled 35 times using the following settings: 94 °C for 1 minute, 60 °C for 1 minute, 72 °C for 1 minute and 30 seconds, followed by a final extension at 72 °C during 5 minutes. Those yeasts that amplified MET2 were identified as *Saccharomyces. Saccharomyces cerevisiae* were identified by positive EcoRI digestion of the MET2 gene. The EcoRI digestion was done on reactions of 50 µL where 20 µL of the PCR product, 5 µL of 10x NEbuffer EcoRI (NEB, Ipswich, MA, USA) and 0.5 µL of the EcoRI enzyme (20 U/µL) (NEB, Ipswich, MA, USA) were used. MET2 gene amplification and resulting restriction fragments were analyzed on LabChip 90 (Caliper Life Sciences, Hopkinton, MA, USA) [2,3].

Those isolates that showed no amplification by PCR of MET2 were classified as non-*Saccharomyces* species, and were then identified by amplification of ITS region, in 25 µL reaction volumes containing 100 ng DNA, 1x reaction buffer (100mM Tris-HCl (pH 8.8 at 25 ºC), 500 mM KCl, 0.8% igepal), 2 mM $MgCl_2$, 0.4 µM of each primer (ITS1: TCC GTA GGT GAA CCT GCG G and ITS4: TCC TCC GCT TAT TGA TAT GC), 0.2 mM of each dNTP (Bioron, Ludwigshafen, Germany) and 2.5 U/µL taq DNA polymerase. After initial denaturation (95 °C for 6 minutes), the reaction mixture was cycled 35 times using the following settings: 94 °C for 40 seconds, 53 °C for 40 seconds, 72 °C for 1 minute, followed by a final extension at 72 °C during 5 minutes. The PCR product was analyzed on LabChip 90 (Caliper Life Sciences, Hopkinton, MA,

USA) and the size of the fragments compared with the values presented on S1 Table 1 [4,5].

S1 Table 1. ITS fragment size and corresponding species (adapted from [4,5])

Species	Amplicon (bp)
Schizosaccharomyces pombe	970
Saccharomyces bayanus<br>*Saccharomyces cerevisiae*<br>*Saccharomyces pastorianus*	880
Candida colliculosa	810
Torulaspora delbrueckii	803
Candida glabrata	800
Hanseniaspora uvarum<br>*Kloeckera apiculata*	760
Saccharomyces ludwigii	758
Zygosaccharomyces baiili	750
Candida boidinii	700
Kluyveromyces thermotolerans	682
Candida tenuis	680
Candida famata<br>*Debarymoces hansenii*	656
Candida zeylanoides	620
Pichia guilliermondii	606
Candida norvegica	580
Candida albicans<br>*Candida tropicalis*	550
Candida parapsilosis	520
Dekkera anómala	514
Brettanomyces bruxellensis<br>*Candida stellata*<br>*Dekkera intermédia*<br>*Pichia membranaefaciens*	500
Issatchenkia orientalis	494
Candida sake<br>*Pichia fermentans*<br>*Pichia kluyveri*	470
Dekkera bruxellensis	459
Metschnikowia pulcherrima	390

Genomic fingerprinting by interdelta region amplification

Saccharomyces cerevisiae were genotyped by electrophoretic profiling of interdelta sequence. DNA amplification was performed recurring to primers d12 (5'-TCA ACA ATG GAA TCC CAA C-3') and d2 (5'-GTG GAT TTT TAT TCC AAC-3'). Thirty microliter of reaction mixture was prepared with 100 ng of DNA, Taq buffer

(67 mM Tris-HCl, 16 mM $(NH_4)_2SO_4$, 0.01% Tween-20), 1.68 µM of each primer, 0.4 mM of each dNTP, 3 mM $MgCl_2$ (Bioron, Ludwigshafen, Germany) and 1.0 U of TaqDNA polymerase (Bioron, Ludwigshafen, Germany). After initial denaturation (95 °C for 2 minutes), the reaction mixture was cycled 35 times using the following settings: 95 °C for 30 seconds, 43.2 °C for 1 minute, 72 °C for 1 minute, followed by a final extension at 72 °C during 10 minutes. PCR products were analyzed using a high throughput automated microfluidic electrophoresis system (Caliper LabChip 90 Electrophoresis System (Caliper Life Sciences, Hopkinton, MA, USA)) and a 96-well plate format, according to the manufacturer's instructions [3,6,7]. The resulting interdelta electrophoretic profiles were imported into BioNumerics 6.5 (Applied Maths, Sint-Martens-Latem, Belgium) and clustered according to their similarities.

Using this method we have grouped the 1789 *S. cerevisiae* in 313 strains that were phenotyped as explained in the S2 Protocol from Supporting information.

References

1. Querol A, Barrio E, Huerta T, Ramón D. Molecular Monitoring of Wine Fermentations Conducted by Active Dry Yeast Strains. Appl Environ Microbiol. 1992;58: 2948–2953.

2. Masneuf I, Aigle M, Dubourdieu D. Development of a polymerase chain reaction/restriction fragment length polymorphism method for Saccharomyces cerevisiae and Saccharomyces bayanus identification in enology. FEMS Microbiol Lett. 1996;138: 239–244.

3. Ribéreau-Gayon P, Dubourdieu D, Donèche B, Lonvaud A. Handbook of Enology: The Microbiology of Wine and Vinifications. Chichester, UK: John Wiley & Sons, Ltd; 2005.

4. Granchi L, Bosco M, Messini A, Vincenzini M. Rapid detection and quantification of yeast species during spontaneous wine fermentation by PCR–RFLP analysis of the rDNA ITS region. J Appl Microbiol. Blackwell Science Ltd; 1999;87: 949–956.

5. Guillamón JM, Sabaté J, Barrio E, Cano J, Querol A. Rapid identification of wine yeast species based on RFLP analysis of the ribosomal internal transcribed spacer (ITS) region. Arch Microbiol. 1998;169: 387–92.

6. Carreto L, Eiriz M, Gomes A, Pereira P, Schuller D, Santos M. Comparative genomics of wild type yeast strains unveils important genome diversity. BMC Genomics. 2008;9: 524.

7. Franco-Duarte R, Mendes I, Gomes AC, Santos MAS, de Sousa B, Schuller D. Genotyping of Saccharomyces cerevisiae strains by interdelta sequence typing using automated microfluidics. Electrophoresis. 2011;32: 1447–1455.
