# Supplementary material for: Exploring the Saccharomyces cerevisiae Volatile Metabolome: Indigenous versus Commercial Strains
Source: PLoS One. 2015 Nov 24;10(11):e0143641. doi: 10.1371/journal.pone.0143641 (PMC4657929; doi:10.1371/journal.pone.0143641)
Supplement: S2 Protocol — (PDF) [file pone.0143641.s004.pdf]

Supporting Information – S2 Protocol

Exploring the *Saccharomyces cerevisiae* volatile metabolome: indigenous versus commercial strains

Zélia Alves[1], André Melo[2,3], Ana Raquel Figueiredo[1,2], Manuel A. Coimbra[1], Ana C. Gomes[2], Sílvia M. Rocha[1, *]

[1]Departament of Chemistry & QOPNA, University of Aveiro, 3810-193 Aveiro, Portugal

[2]Genomics Unit, Biocant – Biotechnology Innovation Center, Parque Tecnológico de Cantanhede, Núcleo 4, Lote 8, 3060-197 Cantanhede, Portugal

[3]Departament of Biology & CESAM, University of Aveiro, 3810-193 Aveiro, Portugal

S2 Protocol – Phenotypic *S. cerevisiae* characterization

High throughput phenomics of the 313 *S. cerevisiae* strains

The strains were grown in YPD agar medium (yeast extract 1% (w/v), glucose 2% (w/v), peptone 2% (w/v), agar 2%, Formedium, Norfolk, UK) for 2 days at 30°C. The obtained biomass for each strain was then used to inoculate 96-wells deep wells plate (NUNC, Rochester, New York, USA) containing 1.75 mL of YPD medium (1% (w/v) yeast extract, 2% (w/v) glucose, 2% (w/v) peptone, Formedium, Norfolk, UK). These plates were sealed with a pre-sterilized breathable sealing film (Axygen Scientific) and incubated at 30°C overnight, with gentle agitation (100 rpm). Afterwards, the cells were counted with a TC10 Automated Cell Counter (BioRad, Hercules, CA, USA) and 10^6 cells were transferred to 1 mL of YPD in a 96 well plate (NUNC, Rochester, New York, USA). Three serial dilutions (10^5, 10^4 and 10^3 cells) were also prepared. It was used a liquid handling station (Sciclone ALH 3000 Workstation – Caliper LifeSciences, Hopkinton, Massachusetts, USA) with a sterile 96-pin tool (VP Scientific, San Diego, CA, USA) to inoculate single well plates (NUNC, Rochester, New York, USA) containing the media of interest. All plates were then incubated at 30°C for 2 days unless noted otherwise. After incubation the strains were scored according to their ability to grow from 0 (no growth in any concentration) to 8 (maximum growth observed in the most diluted concentration) and were clustered using the Pearson correlation and UPGMA clustering (MultiExperiment Viewer 4.7.4).

Carbon source media

Different carbon sources were tested using 0,69% (w/v) YNB without amino acids (Formedium, Norfolk, UK) and 2% (w/v) agar supplemented with different carbon sources - 2% (w/v) fructose, 20% (w/v) fructose, 2% (w/v) maltose, 2% (w/v) sucrose, 2% (w/v) galactose, 2% (w/v) raffinose, 2% (w/v) glucose, 20% (w/v) glucose or 10% (w/v) glucose + 10% (w/v) fructose (Formedium, Norfolk, UK). The use of glycerol as carbon source was also teste in YPG (1% (w/v) yeast extract (Formedium, Norfolk, UK), 2% (w/v) peptone (Formedium, Norfolk, UK), 2% (w/v) agar (Formedium, Norfolk, UK) and 3% (V/V) glycerol);

Nitrogen source media

Three different nitrogen sources were tested using *(i)* 0,69% (w/v) YNB without amino acids, 2% (w/v) glucose and 2% (w/v) agar; *(ii)* 0,19% (w/v) YNB without amino acids and ammonium sulphate, 2% (w/v) glucose and 2% (w/v) agar; and *(iii)* 0,19% (w/v) YNB without amino acids and ammonium sulphate, 2% (w/v) glucose and 2% (w/v) agar, supplemented with 1 amino acid (31% (w/v) proline, 50% (w/v) proline, 27% (w/v) arginine, 50% (w/v) arginine, 13% (w/v) glutamate or 50% (w/v) glutamate) (Formedium, Norfolk, UK);

Drugs and ion tolerance testing media

It was tested growth in YPD agar media supplemented with either NaCl 1M; $CaCl_2$ 0,5 M; $ZnCl_2$ 10 mM; LiCl 50 mM; $CuSO_4$ 10 mM; $CdCl_2$ 500 µM; 0,5 µg/ml of 4-nitroquinoline 1-oxide; 15 mM of caffeine; 0,2 µg/ml of cycloheximide; or 2 mg/ml of paromomycin by sterile filtering stock solutions.

Temperature resistance

The tolerance to different growth temperatures was evaluated with YPD agar medium (yeast extract 1% (w/v), glucose 2% (w/v), peptone 2% (w/v), agar 2%, Formedium, Norfolk, UK) incubated at 12°C (for 7 days), 30°C or 42°C.

Oxidative stress

Oxidative stress tolerance was evaluated with YPD agar (yeast extract 1% (w/v), glucose 2% (w/v), peptone 2% (w/v), agar 2%, Formedium, Norfolk, UK) supplemented with 7 mM of H_2O_2 by sterile filtering a stock solution.

Hydrogen sulphide (H_2S) production

The capacity to produce H_2S was evaluated by plating 10^6 cells to a commercially available BiGGY agar medium (73608 BiGGY Agar, Fluka,St. Louis, Missouri, United States) prepared according to manufacturer's instructions. After 2 days at 25°C, H_2S formation was evaluated based on the varying colony colour, which turn light brown to black if H_2S is formed or remain white if there is no production of H_2S. The yeast scores were based on presence or absence of H_2S (white colonies scored 0 and non-white colonies scored 1).

Ethanol tolerance

Ethanol tolerance was evaluated with YPD agar (yeast extract 1% (w/v), glucose 2% (w/v), peptone 2% (w/v), agar 2%, Formedium, Norfolk, UK) plates supplemented with 12% and 15% (v/v) of ethanol.

Sulfur dioxide (SO_2) tolerance

Sulfur dioxide tolerance was evaluated with YPD agar (yeast extract 1% (w/v), glucose 2% (w/v), peptone 2% (w/v), agar 2%, Formedium, Norfolk, UK) supplemented with 200 mg/L of H_2SO_3.
