# Supplementary material for: Exploring the Saccharomyces cerevisiae Volatile Metabolome: Indigenous versus Commercial Strains
Source: PLoS One. 2015 Nov 24;10(11):e0143641. doi: 10.1371/journal.pone.0143641 (PMC4657929; doi:10.1371/journal.pone.0143641)
Supplement: S1 Table — (PDF) [file pone.0143641.s005.pdf]

Supporting Information – S1 Table

Exploring the *Saccharomyces cerevisiae* metabolomics biodiversity: indigenous versus commercial strains

Zélia Alves[1], André Melo[2,3], Ana Raquel Figueiredo[1,2], Manuel A. Coimbra[1], Ana C. Gomes[2], Sílvia M. Rocha[1, *]

[1]Departament of Chemistry & QOPNA, University of Aveiro, 3810-193 Aveiro, Portugal

[2]Genomics Unit, Biocant – Biotechnology Innovation Center, Parque Tecnológico de Cantanhede, Núcleo 4, Lote 8, 3060-197 Cantanhede, Portugal

[3]Departament of Biology & CESAM, University of Aveiro, 3810-193 Aveiro, Portugal

S1 Table. Volatile metabolites identified by HS-SPME/GC×GC-ToFMS in four *S. cerevisiae* strains under study: BT2453 and BT2652 corresponding to strains isolated from wine, and CSc1 and CSc2, corresponding to commercial yeast strains.

Nº	$^{1}t_R{}^{a}$ (s)	$^{2}t_R{}^{a}$ (s)	Compound	Formula	CAS	RI $_{Calc}$ [c]	RI $_{Lit}$ [d]	BT2453	BT2652	CSc1	CSc2	Ref
								Peak Area[b] (x10^6) and RSD (%)				
			**Acetals**									
1	145	0.432	1,1-Diethoxyethane	$C_6H_{14}O_2$	105-57-7	711	690	1.33 (20)	1.64 (11)	1.00 (17)	1.30 (35)	[1]
2	145	0.456	2,4,5-Trimethyl-1,3-dioxolane	$C_6H_{12}O_2$	3299-32-9	711	690	2.26 (35)	3.04 (9)	1.31 (3)	1.66 (25)	[1]
3	290	0.728	2,2,3-Trimethyloxetane	$C_6H_{12}O$	23120-43-6	917	-	0.22 (18)	-	-	-	
			**Subtotal (GC Peak Area)**					**3.81**	**4.68**	**2.31**	**2.96**	
			**Subtotal (%)**					**0.36**	**0.64**	**0.33**	**0.39**	
			**Acids**									
4	100	3.336	Acetic acid	$C_2H_4O_2$	64-19-7	636	600	63.93 (32)	63.10 (22)	40.84 (8)	1.50 (6)	[2]
5	135	4.344	Propanoic acid	$C_3H_6O_2$	79-09-4	699	700	1.17 (13)	0.90 (17)	-	0.31 (6)	[3]
6	170	3.904	2-Methylpropanoic acid	$C_4H_8O_2$	79-31-2	768	775	12.28 (11)	8.43 (15)	4.49 (15)	5.93 (8)	[4]
7	190	4.640	Butanoic acid	$C_4H_8O_2$	107-92-6	805	821	8.17 (4)	4.02 (8)	2.86 (8)	4.09 (18)	[5]
8	235	4.056	Isovaleric acid	$C_5H_{10}O_2$	503-74-2	858	864	11.36 (18)	7.59 (7)	4.08 (11)	5.93 (12)	[6]
9	245	3.776	2-Methylbutanoic acid	$C_5H_{10}O_2$	116-53-0	869	873	6.27 (26)	5.33 (6)	2.50 (15)	2.82 (12)	[5]
10	365	3.648	Hexanoic acid	$C_6H_{12}O_2$	142-62-1	999	1019	37.94 (13)	9.07 (8)	18.35 (13)	19.46 (19)	[5]
11	545	2.616	Octanoic acid	$C_8H_{16}O_2$	124-07-2	1197	1182	214.92 (6)	141.41 (20)	131.62 (12)	141.76 (17)	[7]
12	615	2.448	Nonanoic acid	$C_9H_{18}O_2$	112-05-0	1279	1280	9.36 (161)	0.39 (49)	-	-	[8]
13	695	2.616	Dec-9-enoic acid	$C_{10}H_{18}O_2$	14436-32-9	1378	1389	13.91 (12)	12.03 (65)	-	-	[9]
14	700	2.184	Decanoic acid	$C_{10}H_{20}O_2$	334-48-5	1378	1380	97.46 (17)	50.06 (8)	63.33 (18)	73.65 (14)	[10]
			**Subtotal (GC Peak Area)**					**476.78**	**302.34**	**268.07**	**255.45**	
			**Subtotal (%)**					**45.47**	**41.48**	**38.51**	**33.44**	
			**Alcohols**									
			Aliphatics									
15	85	0.648	1-Propanol	C_3H_8O	71-23-8	591	574	3.29 (12)	3.45 (9)	2.41 (16)	2.51 (12)	[11]
16	100	0.712	2-Methyl-1-propanol	$C_4H_{10}O$	78-83-1	621	615	11.88 (25)	8.84 (10)	15.61 (11)	12.41 (11)	[12]
17	105	0.568	2-Methyl-2-butanol	$C_5H_{12}O$	75-85-4	631	622	1.87 (67)	2.36 (13)	1.49 (25)	1.38 (50)	[12]
18	115	0.784	1-Butanol	$C_4H_{10}O$	71-36-3	652	646	1.18 (24)	1.38 (19)	0.91 (24)	0.74 (11)	[13]
19	130	0.696	2-Pentanol	$C_5H_{12}O$	6032-29-7	681	685	-	0.04 (19)	-	- (13)	[14]
20	145	0.640	2-Methyl-2-pentanol	$C_6H_{14}O$	590-36-3	721	698	0.47 (82)	0.36 (13)	0.26 (16)	0.35 (8)	[12]
21	150	0.944	3-Methyl-1-butanol	$C_5H_{12}O$	123-51-3	722	706	35.55 (20)	32.61 (16)	33.68 (23)	38.32 (22)	[12]
22	155	0.864	2-Methyl-1-butanol	$C_5H_{12}O$	137-32-6	732	739	-	-	16.93 (5)	16.58 (7)	[2]
23	165	0.720	4-Methyl-2-pentanol	$C_6H_{14}O$	108-11-2	751	758	0.08 (15)	0.06 (14)	0.08 (19)	0.09 (15)	[3]
24	175	0.912	1-Pentanol	$C_5H_{12}O$	71-41-0	772	776	0.40 (10)	-	-	-	[15]
25	180	1.184	3-Methyl-2-buten-1-ol	$C_5H_{10}O$	556-82-1	782	778	0.17 (35)	-	-	-	[8]
26	185	0.632	2,4-Dimethyl-2-pentanol	$C_7H_{16}O$	625-06-9	791	-	0.12 (33)	0.16 (24)	0.12 (2)	0.11 (26)	
27	220	0.672	2-Methyl-2-hexanol	$C_7H_{16}O$	625-23-0	836	-	0.10 (24)	0.09 (18)	0.08 (21)	0.08 (22)	
28	225	0.904	2-Methyl-1-pentanol	$C_6H_{14}O$	105-30-6	842	843	0.56 (7)	-	0.28 (3)	0.29 (3)	[16]

29	260	0.944	1-Hexanol	$C_6H_{14}O$	111-27-3	883	874	0.10 (5)	0.08 (58)	-	-	[17]
30	280	0.792	2-Heptanol	$C_7H_{16}O$	543-49-7	906	904	0.07 (25)	-	0.10 (10)	0.09 (9)	[11]
31	305	0.664	4-Methyl-4-heptanol	$C_8H_{18}O$	598-01-6	932	-	0.53 (29)	0.45 (11)	0.45 (22)	0.48 (9)	
32	305	0.680	2-Methyl-2-heptanol	$C_8H_{18}O$	625-25-2	932	-	0.63 (31)	0.44 (12)	0.46 (20)	0.45 (18)	
33	330	0.768	4-Methyl-2-heptanol	$C_8H_{18}O$	56298-90-9	959	950	4.54 (22)	3.91 (19)	3.15 (6)	3.42 (5)	[18]
34	345	0.960	1-Heptanol	$C_7H_{16}O$	111-70-6	975	974	3.00 (42)	3.71 (9)	0.45 (8)	0.74 (13)	[15]
35	350	0.656	2,3-Dimethyl-2-hexanol	$C_8H_{18}O$	19550-03-9	980	-	6.23 (16)	5.68 (16)	6.21 (6)	6.31 (6)	
36	355	0.624	3-Ethyl-2-methyl-3-pentanol	$C_8H_{18}O$	597-05-7	985	-	0.66 (20)	0.54 (16)	0.59 (13)	0.62 (7)	
37	360	0.760	2-Methyl-2-octen-4-ol	$C_9H_{18}O$	-	990	-	0.36 (22)	-	0.57 (10)	0.29 (19)	
38	380	0.728	C_8 Alcohol (*m/z* 45, 57, 69)	$C_8H_{18}O$	-	1011	-	0.76 (10)	0.78 (15)	0.63 (17)	0.66 (10)	
39	395	0.656	4-Methyl-4-octanol	$C_9H_{20}O$	23418-37-3	1027	-	0.57 (25)	0.43 (20)	0.50 (8)	0.54 (4)	
40	395	0.688	4,5-Dimethyl-3-heptanol	$C_9H_{20}O$	-	1027	-	-	-	0.33 (49)	0.43 (7)	
41	400	0.624	2-Methyl-2-octanol	$C_9H_{20}O$	628-44-4	1032	1033	0.77 (11)	0.83 (15)	0.63 (11)	0.65 (20)	[19]
42	400	0.872	2-Ethyl-1-hexanol	$C_8H_{18}O$	104-76-7	1033	1038	8.29 (20)	6.23 (19)	5.52 (12)	6.05 (2)	[17]
43	430	1.800	2,5-Dimethyl-2,5-hexanediol	$C_8H_{18}O_2$	110-03-2	1065	-	0.66 (32)	0.11 (59)	-	0.40 (16)	
44	435	0.744	C_8 Alcohol (*m/z* 45, 55, 70)	$C_8H_{18}O$	-	1069	-	0.56 (25)	-	-	-	
45	445	0.888	1-Octanol	$C_8H_{18}O$	111-87-5	1075	1079	0.87 (18)	1.11 (15)	1.54 (13)	1.63 (34)	[17]
46	450	0.632	2,4-Dimethyl-3-hexanol	$C_8H_{18}O$	13432-25-2	1085	-	0.28 (40)	0.22 (5)	0.25 (5)	0.17 (5)	
47	455	0.840	2,4-Dimethyl-1-heptanol	$C_9H_{20}O$	-	1090	-	-	-	4.61 (25)	1.89 (14)	
48	460	0.896	2,3,4-Trimethyl-5-hexen-3-ol	$C_9H_{18}O$	28638-29-1	1090	-	1.54 (35)	0.83 (12)	5.36 (22)	0.76 (40)	
49	465	0.768	2-Nonanol	$C_9H_{20}O$	628-99-9	1101	1098	0.22 (9)	-	0.17 (6)	0.20 (14)	[8]
50	465	1.008	2-Nonen-1-ol	$C_9H_{18}O$	31502-14-4	1101	1074	1.43 (22)	-	-	-	[20]
51	480	0.640	4-Ethyl-4-heptanol	$C_9H_{20}O$	597-90-0	1118	-		0.21 (17)	-	-	
52	485	0.648	4-Methyl-4-nonanol	$C_{10}H_{22}O$	23418-38-4	1124	-	1.98 (19)	1.28 (15)	1.02 (10)	1.17 (7)	
53	495	0.616	C_{10} Alcohol (*m/z* 41, 55,59)	$C_{10}H_{22}O$	-	1136	-	-	-	-	0.70 (20)	
54	525	0.616	3,4-Diethyl-3-hexanol	$C_{10}H_{22}O$	19398-78-8	1171	-	7.02 (8)	6.79 (15)	6.10 (12)	5.53 (19)	
55	530	0.720	C_{10} Alcohol (*m/z* 45, 55, 70)	$C_{10}H_{22}O$	-	1177	-	-	0.14 (27)	1.47 (16)	3.95 (11)	
56	545	0.832	1-Nonanol	$C_9H_{20}O$	143-08-8	1195	1179	5.04 (61)	3.01 (21)	3.17 (26)	3.95 (12)	[17]
57	555	0.680	2-Decanol	$C_{10}H_{22}O$	1120-06-5	1207	1209	3.16 (14)	2.32 (12)	1.14 (26)	1.25 (38)	[21]
58	565	0.616	3-Decanol	$C_{10}H_{22}O$	1565-81-7	1218	1197	6.12 (21)	4.93 (32)	5.30 (17)	-	[8]
59	620	0.832	1-Decanol	$C_{10}H_{22}O$	112-30-1	1283	1281	1.96 (16)	2.84 (22)	3.10 (15)	3.08 (23)	[17]
60	650	0.440	2-Butyl-1-octanol	$C_{12}H_{26}O$	3913-02-8	1319	-	0.41 (21)	-	-	-	
			Aromatics									
61	450	1.600	2-Phenylisopropanol	$C_9H_{12}O$	617-94-7	1086	1080	-	-	-	0.12 (13)	[22]
62	480	2.272	2-Phenylethanol	$C_8H_{10}O$	60-12-8	1121	1110	78.54 (20)	30.40 (19)	45.23 (10)	58.52 (2)	[8]
			**Cyclic**									
63	365	0.688	1-Cyclopentyl-1-propanol	$C_8H_{16}O$	-	995	-	-	-	-	0.19 (8)	
			**Subtotal (GC Peak Area)**					**191.93**	**126.64**	**165.07**	**177.18**	
			**Subtotal (%)**					**18.30**	**17.37**	**23.71**	**23.19**	
			**Aldehydes**									
			Aliphatics									
64	85	0.408	Butanal	C_4H_8O	123-72-8	591	614	1.38 (85)	-	1.78 (20)	2.17 (18)	[20]
65	110	0.464	3-Methyl-butanal	$C_5H_{10}O$	590-86-3	641	649	6.65 (33)	6.52 (29)	5.68 (27)	7.44 (19)	[11]
66	115	0.456	2-Methyl-butanal	$C_5H_{10}O$	96-17-3	651	646	3.92 (46)	3.17 (34)	4.04 (12)	4.37 (5)	[23]
67	425	0.736	2-Octenal	$C_8H_{14}O$	2548-87-0	1059	1056	-	2.60 (4)	0.81 (83)	0.70 (9)	[15]

68	605	0.728	2-Decenal	$C_{10}H_{18}O$	3913-81-3	1266	1261	1.60 (12)	-	-	-	[8]
			Aromatics									
69	335	1.288	Benzaldehyde	C_7H_6O	100-52-7	965	964	9.91 (62)	1.65 (19)	3.45 (15)	7.20 (20)	[20]
70	410	1.344	2-Phenylacetaldehyde	C_8H_8O	122-78-1	1044	1043	11.25 (51)	3.22 (10)	5.28 (4)	7.61 (12)	[8]
71	410	1.528	2-Hydroxy benzaldehyde	$C_7H_6O_2$	90-02-8	1044	1041	0.08 (53)	-	-	-	[8]
72	565	1.208	3,5-Dimethyl-benzaldehyde	$C_9H_{10}O$	5779-95-3	1219	-	6.55 (15)	-	1.27 (16)	-	
73	615	1.312	2-Phenyl-2-butenal	$C_{10}H_{10}O$	4411-89-6	1278	1273	0.17 (37)	-	-	-	[24]
			**Subtotal (GC Peak Area)**					**41.50**	**17.16**	**22.32**	**29.49**	
			**Subtotal (%)**					**3.96**	**2.35**	**3.21**	**3.86**	
			**Ketones**									
			Aliphatics									
74	90	0.552	2,3-Butanedione	$C_4H_6O_2$	431-03-8	601	592	-	-	-	0.29 (12)	[2]
75	95	0.448	2-Butanone	C_4H_8O	78-93-3	611	601	-	-	0.50 (3)	0.56 (13)	[20]
76	125	0.504	2-Pentanone	$C_5H_{10}O$	107-87-9	671	686	1.04 (44)	0.95 (22)	0.97 (12)	1.04 (14)	[25]
77	125	0.624	2,3-Pentanedione	$C_5H_8O_2$	600-14-6	671	693	-	0.14 (54)	0.33 (45)	0.31 (15)	[3]
78	140	1.232	3-Hydroxy-2-butanone	$C_4H_8O_2$	513-86-0	702	733	5.43 (77)	3.34 (72)	5.24 (46)	5.29 (17)	[15]
79	150	0.512	4-Methyl-2-pentanone	$C_6H_{12}O$	180-10-1	721	736	1.50 (25)	1.36 (20)	1.27 (14)	1.26 (11)	[3]
80	185	0.576	2-Hexanone	$C_6H_{12}O$	591-78-6	791	787	0.39 (26)	-	0.38 (8)	0.37 (12)	[11]
81	190	0.648	4-Methyl-3-penten-2-one	$C_6H_{10}O$	141-79-7	801	801	0.82 (63)	-	0.37 (9)	0.37 (12)	[12]
82	195	1.216	3-Hydroxy-2-pentanone	$C_5H_{10}O_2$	3142-66-3	807	803	0.13 (45)	0.49 (15)	0.11 (17)	0.14 (23)	[26]
83	220	0.616	2,3-Heptanedione	$C_7H_{12}O_2$	96-04-8	836	-	-	0.07 (15)	-	0.07 (15)	
84	230	0.568	4-Methyl-2-hexanone	$C_7H_{14}O$	105-42-0	848	846	0.31 (48)	0.26 (16)	0.29 (8)	0.29 (10)	[27]
85	230	1.120	4-Hydroxy-4-methyl-2-pentanone	$C_6H_{12}O_2$	123-42-2	848	840	0.25 (135)	-	-	-	[28]
86	240	0.584	5-Methyl-2-hexanone	$C_7H_{14}O$	110-12-3	860	857	0.68 (30)	0.55 (16)	-	-	[29]
87	270	0.600	2-Heptanone	$C_7H_{14}O$	110-43-0	895	895	3.07 (37)	2.46 (52)	4.70 (20)	4.92 (10)	[15]
88	280	0.552	3,4-Dimethyl-2-hexanone	$C_8H_{16}O$	19550-10-8	906	-	0.24 (31)	0.18 (36)	0.20 (14)	0.20 (17)	
89	305	0.560	6-Methyl-2-heptanone	$C_8H_{16}O$	928-68-7	932	958	-	7.84 (19)	9.17 (7)	9.36 (9)	[15]
90	305	1.368	2,5-Hexanedione	$C_6H_{10}O_2$	110-13-4	933	933	0.65 (29)	0.29 (18)	0.26 (18)	0.26 (10)	[10]
91	315	0.584	4-Methyl-2-heptanone	$C_8H_{16}O$	6137-06-0	943	936	15.10 (15)	15.40 (9)	15.00 (9)	15.60 (10)	[27]
92	320	1.040	2,5-Dimethyl-3-hexanone	$C_8H_{16}O$	1888-57-9	948	-	-	0.22 (70)	-	-	
93	330	0.472	2,3-Octanedione	$C_8H_{14}O_2$	585-25-1	958	980	-	0.13 (12)	0.10 (18)	0.11 (13)	[3]
94	330	0.624	4-Methyl-3-hepten-2-one (isomer)	$C_8H_{14}O$	22319-25-1	959	-	0.28 (23)	0.20 (24)	-	-	
95	345	0.568	4-Octanone	$C_8H_{16}O$	589-63-9	974	974	0.24 (19)	0.16 (14)	0.16 (20)	-	[3]
96	350	0.520	2,4-Dimethyl-3-heptanone	$C_9H_{18}O$	18641-71-9	979	-	-	-	0.19 (25)	0.32 (8)	
97	355	0.544	C_9 Ketone (*m/z* 43, 57, 86)	$C_9H_{18}O$	-	985	-	0.49 (32)	0.48 (20)	0.48 (10)	0.65 (32)	
98	360	0.584	C_9 Ketone (*m/z* 57, 72, 99)	$C_9H_{18}O$	-	990	-	7.15 (18)	7.25 (3)	9.92 (13)	7.49 (6)	
99	360	0.664	4-Methyl-3-hepten-2-one (isomer)	$C_8H_{14}O$	22319-25-1	990	-	0.70 (17)	-	0.54 (15)	0.66 (12)	
100	395	0.568	C_9 Ketone (*m/z* 43, 71)	$C_9H_{18}O$	6137-15-1	1027	-	-	-	-	0.10 (8)	
101	400	0.608	2-Methyl-2-octen-4-one	$C_9H_{16}O$	-	1032	-	0.28 (13)	0.26 (8)	0.23 (3)	0.24 (2)	
102	405	0.592	C_9 Ketone (*m/z* 43, 58, 85)	$C_9H_{18}O$	-	1038	-	1.49 (26)	1.28 (16)	1.27 (9)	1.26 (20)	
103	410	0.704	3,3,6-Trimethylhepta-1,5-dien-4-one	$C_{10}H_{16}O$	546-49-6	1043	1062	0.54 (30)	0.37 (6)	0.39 (19)	0.40 (15)	[8]
104	430	0.696	5-Nonen-2-one	$C_9H_{16}O$	27039-84-5	1064	-	0.87 (30)	0.70 (15)	0.46 (1)	0.63 (19)	
105	440	0.576	5-Nonanone	$C_9H_{18}O$	502-56-7	1074	1073	0.60 (34)	0.34 (36)	0.51 (19)	-	[3]
106	450	0.584	3,5-Dimethyl-2-octanone	$C_{10}H_{20}O$	19781-14-7	1085	-	0.12 (24)	0.10 (8)	0.08 (14)	0.10 (11)	
107	455	0.624	2-Nonanone	$C_9H_{18}O$	821-55-6	1090	1093	3.94 (35)	3.03 (21)	4.54 (14)	6.19 (1)	[20]

108	475	0.752	2,6-Dimethyl-2,5-heptadien-4-one	$C_9H_{14}O$	504-20-1	1113	-	7.55 (13)	6.39 (7)	5.68 (11)	6.08 (10)	
109	480	0.560	4-Decanone	$C_{10}H_{20}O$	624-16-8	1118	-	0.67 (12)	0.59 (17)	0.64 (12)	0.57 (2)	
110	540	0.608	3-Decanone	$C_{10}H_{20}O$	928-80-3	1189	1186	2.32 (34)	0.10 (13)	0.12 (29)	0.12 (20)	[8]
111	580	0.640	2-Methyl-2-nonen-4-one	$C_{10}H_{18}O$	2903-23-3	1236	1215	-	-	0.43 (20)	0.43 (20)	[30]
112	630	0.640	2-Undecanone	$C_{11}H_{22}O$	112-12-9	1295	1291	1.20 (16)	-	-	-	[17]
			Aromatics									
113	430	1.288	1-Phenylethanone	C_8H_8O	98-86-2	1065	1067	2.64 (12)	0.57 (11)	-	-	[15]
114	490	1.248	1-Phenylpropan-2-one	$C_9H_{10}O$	103-79-7	1131	1124	0.15 (1)	-	-	-	[31]
115	495	0.968	1-(2-Methylphenyl)ethanone	$C_9H_{10}O$	577-16-2	1136	1139	0.09 (21)	-	-	-	[32]
116	540	1.192	1-(4-Methylphenyl)ethanone	$C_9H_{10}O$	122-00-9	1190	1182	0.08 (25)	0.06 (20)	-	-	[8]
117	575	1.112	1-Phenyl-2-butanone	$C_{10}H_{12}O$	1007-32-5	1231	-	0.10 (6)	0.05 (4)	-	-	
118	625	1.352	4-Phenyl-3-buten-2-one	$C_{10}H_{10}O$	1896-62-4	1290	1330	0.10 (41)	-	-	-	[33]
119	650	1.208	1-(3,4-Dimethyphenyl)-ethanone	$C_{10}H_{12}O$	03637-01-2	1320	-	0.20 (21)	0.11 (30)	-	-	
			Cyclic									
120	270	0.808	Cyclohexanone	$C_6H_{10}O$	108-94-1	895	895	0.41 (21)	0.31 (12)	0.40 (17)	0.36 (8)	[10]
			**Subtotal (GC Peak Area)**					**61.79**	**56.01**	**64.93**	**66.07**	
			**Subtotal (%)**					**5.89**	**7.68**	**9.33**	**8.65**	
			**Terpenic compounds**									
121	365	0.504	β-Myrcene	$C_{10}H_{16}$	123-35-3	995	986	0.50 (9)	0.27 (15)	0.20 (14)	0.24 (17)	[34]
122	385	0.504	α-Terpinene	$C_{10}H_{16}$	99-86-5	1016	1021	0.68 (147)	-	-	-	[35]
123	400	0.504	Limonene	$C_{10}H_{16}$	138-86-3	1032	1027	0.22 (29)	0.91 (14)	0.38 (14)	0.46 (6)	[35]
124	415	0.528	β-Ocimene	$C_{10}H_{16}$	13877-91-3	1048	1042	0.39 (40)	0.25 (16)	0.13 (23)	0.18 (11)	[35]
125	415	0.712	Dihydromyrcenol	$C_{10}H_{20}O$	53219-21-9	1048	1076	-	0.09 (37)	-	0.15 (13)	[36]
126	425	0.520	γ-Terpinene	$C_{10}H_{16}$	99-85-4	1058	1061	0.54 (145)	-	-	-	[35]
127	440	0.648	6,10-Dihydromyrcenol	$C_{10}H_{22}O$	18479-58-8	1074	1072	0.87 (14)	0.11 (18)	0.10 (4)	0.11 (15)	[37]
128	455	0.528	α-Terpinolene	$C_{10}H_{16}$	586-62-9	1090	1093	1.31 (155)	0.11 (17)	-	-	[35]
129	465	0.816	Linalool	$C_{10}H_{18}O$	78-70-6	1101	1107	21.43 (25)	6.26 (6)	3.59 (12)	4.24 (4)	[35]
130	545	0.920	α-Terpineol	$C_{10}H_{18}O$	98-55-5	1195	1206	2.19 (3)	1.70 (16)	0.67 (14)	0.88 (14)	[38]
131	585	1.016	Nerol	$C_{10}H_{18}O$	106-25-2	1237	1242	0.55 (26)	0.48 (6)	-	0.50 (16)	[35]
132	590	1.024	Isogeraniol	$C_{10}H_{18}O$	5944-20-7	1248	1248	2.44 (87)	-	-	-	[39]
133	605	1.048	Geraniol	$C_{10}H_{18}O$	106-24-1	1266	1265	4.00 (6)	1.26 (19)	0.65 (17)	0.94 (10)	[36]
134	610	0.848	Citral	$C_{10}H_{16}O$	5392-40-5	1272	1240	0.26 (17)	0.22 (17)	-	-	[40]
135	700	0.704	Geranyl acetate	$C_{12}H_{20}O_2$	105-87-3	1382	1382	0.25 (22)	-	-	-	[41]
136	760	0.568	β-Farnesene	$C_{15}H_{24}$	28973-97-9	1461	1465	0.48 (56)	0.47 (21)	0.36 (18)	0.43 (19)	[38]
137	795	0.608	α-Farnesene	$C_{15}H_{24}$	502-61-4	1508	1505	0.46 (8)	-	-	0.20 (16)	[42]
138	835	0.840	Nerolidol	$C_{15}H_{26}O$	40716-66-3	1566	1568	3.44 (22)	2.35 (7)	1.30 (18)	1.60 (11)	[43]
			**Subtotal (GC Peak Area)**					**40.02**	**14.47**	**7.40**	**9.93**	
			**Subtotal (%)**					**3.82**	**1.99**	**1.06**	**1.30**	
			**Esters**									
			Aliphatics									
139	75	0.648	Propyl formate	$C_4H_8O_2$	110-74-7	571	-	-	-	2.59 (9)	2.29 (3)	
140	100	0.456	Ethyl acetate	$C_4H_8O_2$	141-78-6	621	608	12.72 (14)	16.11 (34)	13.43 (16)	12.03 (2)	[44]
141	135	0.472	Ethyl propanoate	$C_5H_{10}O_2$	105-37-3	691	714	1.46 (28)	1.36 (19)	0.84 (10)	0.91 (4)	[8]
142	140	0.488	Propyl acetate	$C_5H_{10}O_2$	109-60-4	701	712	-	-	0.16 (32)	0.15 (7)	[10]
143	165	0.464	Ethyl isobutyrate	$C_6H_{12}O_2$	97-62-1	751	747	0.32 (70)	0.19 (24)	0.12 (14)	0.13 (16)	[45]

144	175	0.496	Isobutyl acetate	$C_6H_{12}O_2$	110-19-0	771	770	-		0.47	(45)	0.88	(20)	0.81	(9)	[14]
145	185	0.560	Isopentyl formate	$C_6H_{12}O_2$	110-45-2	791	792	0.28	(24)	0.24	(17)	0.20	(8)	0.24	(15)	[46]
146	195	0.520	Ethyl butyrate	$C_6H_{12}O_2$	105-54-4	806	800	3.69	(20)	3.19	(28)	2.46	(14)	2.24	(8)	[8]
147	200	0.472	1,1-Dimethylpropyl acetate	$C_7H_{14}O_2$	625-16-1	812	-	-		0.38	(20)	0.39	(6)	0.29	(29)	
148	205	1.152	Ethyl 2-hydroxypropanoate	$C_5H_{10}O_3$	97-64-3	819	-	0.04	(18)	-		-		-		
149	230	0.624	Ethyl 2-butenoate	$C_6H_{10}O_2$	10544-63-5	848	863	0.09	(29)	0.06	(20)	-		0.06	(9)	[47]
150	235	0.496	Ethyl 2-methylbutanoate	$C_7H_{14}O_2$	7452-79-1	854	846	-		0.14	(25)	-		-		[48]
151	255	0.552	2/3-Methylbutyl acetate	C_7-$H_{14}O_2$	624-41-9 / 123-92-2	877	880	12.89	(5)	12.80	(50)	13.10	(18)	12.86	(10)	[10]
152	280	0.536	Ethyl pentanoate	$C_7H_{14}O_2$	539-82-2	906	898	0.06	(21)	-		-		-		[8]
153	290	0.520	2-Hexyl acetate	$C_8H_{16}O_2$	142-92-7	916	1008	-		-		0.11	(13)	0.11	(37)	[8]
154	325	1.112	Methyl 2-hydroxy-4-methylpentanoate	$C_7H_{14}O_3$	40348-72-9	954	-	-		-		-		0.30	(7)	
155	340	0.536	Pentyl propionate	$C_8H_{16}O_2$	624-54-4	969	972	0.38	(17)	0.18	(37)	-		0.41	(33)	[8]
156	345	0.664	Methyl 2,4-dimethyl-3-oxopentanoate	$C_8H_{14}O_3$	-	974	-	4.93	(19)	6.67	(10)	5.92	(6)	6.66	(10)	
157	345	2.640	1,3-Propanodiol diacetate	$C_7H_{12}O_4$	628-66-0	976	-	0.51	(18)	1.02	(21)	-		-		
158	365	0.544	Butyl butanoate	$C_8H_{16}O_2$	109-21-7	995	993	0.09	(28)	0.07	(17)	-		-		[8]
159	370	0.552	Ethyl hexanoate	$C_8H_{16}O_2$	123-66-0	1001	996	14.00	(3)	15.61	(10)	14.90	(5)	13.04	(3)	[8]
160	400	0.720	Butyl pivalate	$C_9H_{18}O_2$	5129-37-3	1032	-	-		-		0.08	(32)	-		
161	425	0.528	3-Methylbutyl butyrate	$C_9H_{18}O_2$	106-27-4	1059	1060	-		0.06	(47)	-		0.04	(22)	[8]
162	440	0.920	Octyl formate	$C_9H_{18}O_2$	112-32-3	1075	1060	-		-		1.54	(13)	1.23	(18)	[8]
163	460	0.560	Ethyl heptanoate	$C_9H_{18}O_2$	106-30-9	1095	1095	0.63	(19)	0.61	(9)	0.39	(20)	0.37	(27)	[8]
164	475	0.584	Heptyl acetate	$C_9H_{18}O_2$	112-06-1	1113	1113	-		0.17	(41)	-		-		[8]
165	510	0.536	Isobutyl hexanoate	$C_{10}H_{20}O_2$	105-79-3	1154	-	0.10	(31)	0.07	(2)	-		0.06	(28)	
166	540	0.616	Ethyl 7-octenoate	$C_{10}H_{18}O_2$	35194-38-8	1189	1186	0.59	(18)	0.54	(15)	-		-		[49]
167	545	0.640	Ethyl octanoate	$C_{10}H_{20}O_2$	106-32-1	1201	1195	54.29	(26)	66.64	(25)	5.18	(18)	61.62	(28)	[8]
168	595	0.552	3-Methylbutyl hexanoate	$C_{11}H_{22}O_2$	2198-61-0	1254	1250	0.97	(4)	0.59	(11)	0.61	(12)	0.62	(9)	[10]
169	605	0.944	C_{11} Ester (*m/z* 42, 43, 113)	$C_{11}H_{22}O_2$	-	1266	-	0.28	(14)	-		-		-		
170	630	0.560	Propyl octanoate	$C_{11}H_{22}O_2$	624-13-5	1295	1290	2.29	(34)	2.26	(4)	-		-		[3]
171	630	0.576	Ethyl nonanoate	$C_{11}H_{22}O_2$	123-29-5	1295	1294	2.51	(13)	3.64	(20)	3.22	(17)	2.63	(20)	[10]
172	675	0.552	Butyl octanoate	$C_{12}H_{24}O_2$	589-75-3	1351	1387	0.60	(26)	0.31	(31)	0.28	(26)	0.30	(20)	[50]
173	690	0.848	Butoxyethanol acetate	$C_{10}H_{20}O_4$	124-17-4	1364	1366	0.21	(2)	-		-		-		[51]
174	700	0.832	2-Methyl, 3-hydroxy-2,4,4-trimethylpentyl propanoate	$C_{12}H_{24}O_3$	-	1382	1387	0.64	(11)	-		-		0.32	(19)	[52]
175	705	0.632	Ethyl dec-9-enoate	$C_{12}H_{22}O_2$	67233-91-4	1388	1387	-		-		14.81	(7)	-		[49]
176	710	0.592	Ethyl decanoate	$C_{12}H_{24}O_2$	110-38-3	1395	1394	34.69	(15)	13.17	(24)	43.47	(20)	67.08	(14)	[8]
177	720	0.608	Decyl acetate	$C_{12}H_{24}O_2$	112-17-4	1408	1408	0.32	(12)	-		-		-		[53]
178	750	0.576	3-Methylbutyl octanoate	$C_{13}H_{26}O_2$	2035-99-6	1447	1444	3.41	(16)	2.11	(39)	1.93	(25)	2.00	(19)	[44]
179	780	0.608	Propyl decanoate	$C_{13}H_{26}O_2$	30673-60-0	1488	1493	0.59	(15)	-		-		0.15	(27)	[46]
180	820	0.616	2-Methylpropyl decanoate	$C_{14}H_{28}O_2$	30673-38-2	1544	1545	0.29	(34)	-		-		-		[54]
181	855	0.624	Ethyl dodecanoate	$C_{14}H_{28}O_2$	106-33-2	1594	1595	7.99	(29)	3.88	(30)	2.69	(11)	3.81	(41)	[8]
182	865	0.704	Dodecyl acetate	$C_{14}H_{28}O_2$	112-66-3	1608	1609	0.56	(26)	-		-		-		[55]
183	890	0.688	3-Methylbutyl decanoate	$C_{15}H_{30}O_2$	-	1641	1647	-		0.59	(6)	0.72	(25)	0.81	(22)	[56]
			Aromatics													
184	460	1.056	Methyl benzoate	$C_8H_8O_2$	93-58-3	1096	1091	4.19	(9)	2.49	(2)	2.36	(11)	2.74	(16)	[8]
185	520	1.112	Benzyl acetate	$C_9H_{10}O_2$	140-11-4	1166	1163	0.25	(23)	-		-		-		[8]
186	525	0.944	Ethyl benzoate	$C_9H_{10}O_2$	93-89-0	1172	1170	0.12	(2)	0.08	(20)	-		-		[8]

187	590	1.000	Ethylphenyl acetate	$C_{10}H_{12}O_2$	101-97-3	1248	1244	0.25 (18)	-	0.07 (18)	0.12 (45)	[10]
188	600	1.040	2-Phenylethyl acetate	$C_{10}H_{12}O_2$	103-45-7	1260	1256	4.34 (3)	1.43 (15)	2.37 (32)	3.11 (40)	[8]
189	675	0.960	Ethyl 3-phenylpropanoate	$C_{11}H_{14}O_2$	2021-28-5	1351	1353	0.06 (16)	-	-	-	[57]
190	675	1.056	Methyl 3,4-dimethylbenzoate	$C_{10}H_{12}O_2$	38404-42-1	1351	1353	0.37 (14)	0.24 (19)	0.16 (8)	0.19 (6)	[8]
191	680	0.952	2-Phenylethyl propionate	$C_{11}H_{14}O_2$	122-70-3	1357	1350	0.05 (41)	-	-	-	[8]
192	745	0.936	2-Phenylethyl butanoate	$C_{12}H_{16}O_2$	103-52-6	1441	1439	0.06 (7)	-	-	-	[8]
			**Subtotal (GC Peak Area)**					**172.09**	**157.38**	**134.98**	**199.75**	
			**Subtotal (%)**					**16.41**	**21.59**	**19.39**	**26.14**	
			**Ethers**									
193	100	0.376	2-Ethoxy-2-methylpropane	$C_6H_{14}O$	637-92-3	621	620	2.06 (5)	2.17 (12)	3.17 (32)	2.89 (44)	[58]
194	120	0.392	2-Methoxy-2-methylbutane	$C_6H_{14}O$	994-05-8	661	642	0.21 (9)	0.38 (11)	0.34 (38)	0.32 (50)	[12]
195	135	0.392	2-Methyl-2-(1-methylethoxy)propane	$C_7H_{16}O$	17348-59-3	691	-	0.24 (57)	0.31 (44)	0.28 (35)	0.26 (49)	
196	305	0.480	1-Butoxypent-2-ene	$C_9H_{18}O$	-	932	-	0.17 (9)	-	-	-	
197	345	0.464	1-(1-Ethoxyethoxy)pentane	$C_9H_{20}O_2$	13442-89-2	974	977	0.25 (46)	0.23 (38)	0.15 (21)	0.19 (24)	[1]
198	470	0.536	2,5-Dimethoxy-2,5-dimethylhexane	$C_{10}H_{22}O_2$	53273-13-5	1107	-	0.06 (19)	0.07 (65)	-	0.03 (10)	
			**Subtotal (GC Peak Area)**					**2.99**	**3.15**	**3.94**	**3.70**	
			**Subtotal (%)**					**0.28**	**0.43**	**0.57**	**0.48**	
			**Furan-type compounds**									
199	165	0.408	Tetrahydro-2,2,5,5-tetramethylfuran	$C_8H_{16}O$	15045-43-9	751	-	3.16 (39)	3.39 (19)	3.64 (11)	3.58 (33)	
200	170	0.752	2-Vinylfuran	C_6H_6O	1487-18-9	762	761	0.05 (11)	-	-	-	[24]
201	200	0.920	Dihydro-2-methyl-3-furanone	$C_5H_8O_2$	3188-00-9	813	804	0.15 (48)	-	-	-	[10]
202	290	1.472	1-(2-Furanyl)-ethanone	$C_6H_6O_2$	1192-62-7	917	917	0.17 (50)	-	-	-	[59]
203	370	0.712	(5-Methyl-2-furyl)methanethiol	C_6H_8OS	59303-05-8	1001	995	0.97 (37)	-	-	-	[60]
204	465	0.464	2,2,4,4-Tetramethyltetrahydrofuran	$C_8H_{16}O$	3358-28-9	1101	-	0.22 (52)	0.20 (40)	0.21 (22)	0.15 (20)	
205	575	1.256	3-Phenylfuran	$C_{10}H_8O$	13679-41-9	1231	1228	0.26 (8)	0.14 (16)	0.07 (34)	0.06 (39)	[29]
206	580	0.632	2,2,3,3,4,4-Hexamethyltetrahydrofuran	$C_{10}H_{20}O$	-	1236	-	0.82 (4)	-	-	-	
207	600	1.360	5-Butyldihydrofuran-2-one	$C_8H_{14}O_2$	104-50-7	1260	1260	0.08 (27)	-	-	-	[10]
208	655	0.784	2,2,4-Trimethyl-5-(2,2-dimethylpropyl)-3-furanone	$C_{12}H_{20}O_2$	-	1326	-	1.20 (13)	-	0.66 (15)	0.76 (12)	
			**Subtotal (GC Peak Area)**					**7.08**	**3.72**	**4.57**	**4.56**	
			**Subtotal (%)**					**0.68**	**0.51**	**0.66**	**0.60**	
			**Hydrocarbons**									
			Aliphatics									
209	170	0.384	2,3-Dimethylhexane	C_8H_{18}	584-94-1	761	758	-	0.37 (10)	-	-	[27]
210	225	0.408	2,4-Dimethyl-1-heptene	C_9H_{18}	19549-87-2	842	836	1.14 (98)	1.16 (11)	0.35 (15)	0.44 (20)	[20]
211	245	0.384	4-Methyloctane	C_9H_{20}	2216-34-4	865	864	-	0.65 (68)	-	-	[61]
212	280	0.400	Nonane	C_9H_{20}	111-84-2	906	900	-	0.06 (19)	0.19 (8)	0.15 (18)	[20]
213	335	0.408	2-Methylnonane	$C_{10}H_{22}$	871-83-0	964	969	-	0.29 (15)	0.21 (8)	0.14 (12)	[20]
214	370	0.400	Decane	$C_{10}H_{22}$	124-18-5	1000	1000	-	0.16 (20)	0.27 (36)	0.22 (9)	[62]
215	405	0.432	3-Undecene	$C_{11}H_{22}$	821-97-6	1037	-	0.16 (15)	-	0.14 (6)	0.16 (15)	
216	445	0.424	7-Methyl-2-decene	$C_{11}H_{22}$	74630-23-2	1079	-	0.37 (35)	0.88 (1)	0.31 (51)	0.23 (15)	
217	455	0.480	C_{11} Hydrocarbon (*m/z* 41,67, 109)	$C_{11}H_{22}$	-	1090	-	-	-	0.14 (35)	0.21 (16)	
218	465	0.424	Undecane	$C_{11}H_{24}$	1120-21-4	1101	1101	-	-	0.90 (13)	-	[17]
219	520	0.424	2-Methylundecane	$C_{12}H_{26}$	7045-71-8	1165	1165	0.10 (7)	-	0.15 (18)	0.11 (10)	[27]
220	555	0.432	Dodecane	$C_{12}H_{26}$	112-40-3	1206	1201	0.80 (25)	0.80 (44)	0.97 (13)	0.71 (4)	[17]

221	620	0.424	Tridecane	$C_{13}H_{28}$	629-50-5	1301	1301	0.43	(8)	0.32	(3)	0.39	(15)	0.35	(11)	[15]
222	790	0.480	Pentadecane	$C_{15}H_{32}$	629-62-9	1501	1500	0.39	(18)	0.43	(10)	0.40	(36)	0.37	(11)	[62]
223	860	0.512	Hexadecane	$C_{16}H_{34}$	544-76-3	1601	1600	-		0.41	(5)	-		0.42	(5)	[62]
			Aromatics													
224	250	0.576	1,3-Dimethylbenzene	C_8H_{10}	108-38-3	871	874	1.21	(8)	-		1.25	(9)	1.15	(16)	[3]
225	270	0.616	1,2-Dimethylbenzene	C_8H_{10}	95-47-6	895	908	1.42	(6)	1.55	(17)	1.37	(12)	1.23	(12)	[63]
226	270	0.728	Ethenylbenzene	C_8H_8	100-42-5	895	891	1.72	(20)	1.41	(31)	-		-		[10]
227	390	0.656	1,2,3-Trimethylbenzene	C_9H_{12}	526-73-8	1021	1022	1.06	(9)	1.17	(23)	1.21	(15)	1.05	(11)	[64]
228	450	0.616	1-Ethyl-2,3-dimethylbenzene	$C_{10}H_{14}$	933-98-2	1085	1094	-		0.04	(32)	-		-		[27]
229	455	0.704	1-Methyl-4-(1-methylethenyl)-benzene	$C_{10}H_{12}$	1195-32-0	1090	1090	-		-		0.08	(13)	-		[65]
230	540	1.104	Naphthalene	$C_{10}H_8$	91-20-3	1190	1179	0.52	(16)	0.52	(17)	0.55	[8]	0.86	(58)	[8]
231	600	0.520	1,3-bis(1,1-Dimethylethyl)benzene	$C_{14}H_{22}$	1014-60-4	1259	-	5.00	(10)	5.41	(9)	4.90	(10)	4.06	(10)	
232	700	1.136	Biphenyl	$C_{12}H_{10}$	92-52-4	1383	1385	0.26	(12)	0.21	(19)	0.15	(14)	0.15	(18)	[16]
			Cyclics													
233	495	0.904	1,2-Dimethyl-1-pentyl-cyclopropane	$C_{10}H_{20}$	62238-04-4	1136	-	-		-		6.18	(5)	-		
234	500	0.864	1-Methylpentyl cyclopropane	C_9H_{18}	6976-28-9	1142	-	20.63	(26)	20.57	(3)	-		-		
235	640	0.448	1-Butyl-2-propyl-cyclopentane	$C_{12}H_{24}$	62199-50-2	1307	-	-		-		-		0.08	(14)	
			**Subtotal (GC Peak Area)**					**35.22**		**36.42**		**20.10**		**12.08**		
			**Subtotal (%)**					**3.36**		**5.00**		**2.89**		**1.58**		
			**Pyrans**													
236	275	0.488	2,2,4,6-Tetramethyl-2H-pyran	$C_9H_{14}O$	-	901	-	-		0.21	(11)	-		-		
237	295	0.512	2,2,6-Trimethyl-4-methylene-2H-pyran	$C_9H_{14}O$	-	922	-	0.14	(17)	0.15	(3)	-		-		
238	340	0.864	2,5-Dimethyl-2-hydroxymethyltetrahydropyran	$C_8H_{16}O_2$	-	969	-	1.13	(13)	1.21	(5)	1.01	(15)	1.09	(5)	
			**Subtotal (GC Peak Area)**					**1.27**		**1.56**		**1.01**		**1.09**		
			**Subtotal (%)**					**0.12**		**0.21**		**0.14**		**0.14**		
			**Pyrazines**													
239	295	0.824	Ethylpyrazine	$C_6H_8N_2$	13925-00-3	922	920	0.10	(32)	-		-		-		[8]
240	295	0.856	2,3-Dimethylpyrazine	$C_6H_8N_2$	5910-89-4	922	920	0.08	(28)	-		-		-		[25]
241	370	0.776	2-Ethyl-5-methylpyrazine	$C_7H_{10}N_2$	13360-64-0	1001	1000	1.05	(29)	0.13	(64)	-		0.11	(46)	[8]
242	375	0.784	Trimethylpyrazine	$C_7H_{10}N_2$	14667-55-1	1006	999	0.66	(17)	0.10	(38)	0.16	(16)	0.16	(31)	[29]
243	390	0.952	2-Ethenyl-5-methylpyrazine	$C_7H_8N_2$	13925-08-1	1022	1022	0.49	(9)	-		-		-		[8]
244	420	0.704	2-Methyl-3-isopropylpyrazine	$C_8H_{12}N_2$	15986-81-9	1053	1056	0.35	(20)	0.09	(27)	-		0.08	(36)	[66]
245	445	0.712	3-Ethyl-2,5-dimethylpyrazine	$C_8H_{12}N_2$	13360-65-1	1080	1079	1.33	(36)	-		-		-		[67]
246	515	0.672	3,5-Diethyl-2-methylpyrazine	$C_9H_{14}N_2$	18138-05-1	1160	1160	0.25	(5)	-		-		-		[68]
			2,5-dimethyl-3-(2-													[69]
247	555	0.648	methylpropyl)pyrazine	$C_{10}H_{16}N_2$	32736-94-0	1207	1207	0.08	(30)	-		-		-		[70]
248	600	0.688	2-Butyl-3,5-dimethylpyrazine	$C_{10}H_{16}N_2$	50888-63-6	1260	1254	0.14	(16)	-		-		-		[71]
			**Subtotal (GC Peak Area)**					**4.51**		**0.32**		**0.16**		**0.36**		
			**Subtotal (%)**					**0.43**		**0.04**		**0.02**		**0.05**		
			**S-compounds**													
249	155	0.600	Methyldisulfide	$C_2H_6S_2$	624-92-0	731	738	0.17	(28)	0.09	(25)	-		0.07	(11)	[72]
250	285	0.952	1,3-Oxathiane	C_4H_8OS	-	912	-	2.59	(18)	1.27	(14)	0.74	(16)	0.80	(14)	
251	360	1.168	Dihydro-2-methylthiophen-3(2H)-one	C_5H_8OS	13679-85-1	991	994	0.87	(80)	1.19	(25)	0.46	(20)	0.53	(13)	[25]
252	360	2.200	3-(Methylthio)propanol	$C_4H_{10}OS$	505-10-2	987	978	5.30	(5)	2.37	(56)	-		-		[2]

253	385	1.536	2-Acetylthiazole	C_5H_5NOS	24295-03-2	1017	1014	0.27 (6)	-	-	-	[8]
254	485	0.968	3-(Methylthio)propyl acetate	$C_6H_{12}O_2S$	16630-55-0	1125	1123	0.13 (26)	-	-	-	[40]
255	485	1.640	3-Methyl-2-thiophenecarboxaldehyde	C_6H_6OS	5834-16-2	1126	1133	0.08 (11)	-	-	-	[25]
256	545	1.256	Benzothiofuran	C_8H_6S	95-15-8	1196	1172	0.09 (18)	-	-	-	[73]
257	575	1.648	Benzothiazole	C_7H_5NS	95-16-9	1231	1223	0.14 (14)	0.10 (34)	-	-	[20]
			**Subtotal (GC Peak Area)**					**9.65**	**5.02**	**1.20**	**1.39**	
			**Subtotal (%)**					**0.92**	**0.69**	**0.17**	**0.18**	
			**Total**					**1048.64**	**728.89**	**696.06**	**764.00**	
			**Number of identified compounds**					**210**	**170**	**156**	**172**	

[a] Retention times for first (^1t_R) and second (^2t_R) dimensions in seconds.

[b] Mean of three independent assays (n=3).

[c] Retention Index obtained through the modulated chromatogram.

[d] RI, Retention Index reported in the literature for Equity-5 column or equivalents:

1. Perestrelo R, Barros AS, Câmara JS, Rocha SM. In-depth search focused on furans, lactones, volatile phenols, and acetals as potential age markers of Madeira wines by comprehensive two-dimensional gas chromatography with time-of-flight mass spectrometry combined with solid phase microextraction. J Agric Food Chem. 2011;59: 3186–3204.
2. Rychlik M, Schieberle P, Grosch W. Compilation of odour thresholds, odour qualities and retention indices of key food odorants. Deutsche Forschungsanstalt für Lebensmittelchemie and Institut für Lebensmittelchemie der Technischen Universität München; 1998.
3. Engel E, Ratel J. Correction of the data generated by mass spectrometry analyses of biological tissues: Application to food authentication. J Chromatogr A. 2007;1154: 331–341.
4. Kotseridis Y, Baumes R. Identification of impact odorants in Bordeaux red grape juice, in the commercial yeast used for its fermentation, and in the produced wine. J Agric Food Chem. 2000;48: 400–406.
5. Schnermann P, Schieberle P. Evaluation of Key Odorants in Milk Chocolate and Cocoa Mass by Aroma Extract Dilution Analyses. J Agric Food Chem. 1997;45: 867–872.
6. Figuérédo G, Cabassu P, Chalchat JC, Pasquier B. Studies of Mediterranean oregano populations. VIII - Chemical composition of essential oils of oreganos of various origins. Flavour Fragr J. 2006;21: 134–139.
7. Wu S, Zorn H, Krings U, Berger RG. Volatiles from submerged and surface-cultured beefsteak fungus, Fistulina hepatica. Flavour Fragr J. 2007;22: 206–213.
8. Adams R. Identification of essential oil components by gas chromatography/mass spectrometry. Carol Stream, IL: Allured Publishing Corporation; 1995.
9. Tret'yakov. Retention Data. NIST Mass Spectrometry Data Center [Internet]. 2007.
10. Pino JA, Mesa J, Muñoz Y, Pilar Martí M, Marbot R. Volatile components from Mango (Mangifera indica L.) cultivars. J Agric Food Chem. 2005;53: 2213–2223.
11. Rembold H, Wallner P, Nitz S, Kollmannsberger H, Drawert F. Volatile components of chickpea (Cicer arietinum L.) seed. J Agric Food Chem. American Chemical Society; 1989;37: 659–662.
12. Rocha SM, Freitas R, Cardoso P, Santos M, Martins R, Figueira E. Exploring the potentialities of comprehensive two-dimensional gas chromatography coupled to time of flight mass spectrometry to distinguish bivalve species: Comparison of two clam species (Venerupis decussata and Venerupis philippinarum). J Chromatogr A. Elsevier B.V.; 2013;1315: 152–61.
13. Isidorov V, Purzyńska A, Modzelewska A, Serowiecka M. Distribution coefficients of aliphatic alcohols, carbonyl compounds and esters between air and Carboxen/polydimethylsiloxane fiber coating. Anal Chim Acta. 2006;560: 103–109.
14. Guichard E, Michel S. Comparison of the relative quantities of aroma compounds found in fresh apricot (Prunus armeniaca) from six different varieties. Z Leb Unters Forsch. 1988;186: 301–307.
15. Salvador AC, Baptista I, Barros AS, Gomes NCM, Cunha A, Almeida A, et al. Can volatile organic metabolites be used to simultaneously assess microbial and mite contamination level in cereal grains and coffee beans? PLoS One. 2013;8: e59338.
16. Leffingwell JC, Alford ED. Volatile constituents of Perique tobacco. JEnvironAgricultFood Chem. 2005;4: 1–16.
17. Silva I, Rocha SM, Coimbra M a, Marriott PJ. Headspace solid-phase microextraction combined with comprehensive two-dimensional gas chromatography time-of-flight mass spectrometry for the determination of volatile compounds from marine salt. J Chromatogr A. Elsevier B.V.; 2010;1217: 5511–21.
18. Konig WA, Joulain D, Hochmuth DH. GC/MS Library: Terpenoids and Related Constituents of Essential Oils [Internet]. 2006. Available: http://www.massfinder.com/mfterpenoids.htm
19. Tognolini M, Barocelli E, Ballabeni V, Bruni R, Bianchi A, Chiavarini M, et al. Comparative screening of plant essential oils: Phenylpropanoid moiety as basic core for antiplatelet activity. Life Sci. 2006;78: 1419–1432.
20. Caldeira M, Barros a. S, Bilelo MJ, Parada A, Câmara JS, Rocha SM. Profiling allergic asthma volatile metabolic patterns using a headspace-solid phase microextraction/gas chromatography based methodology. J Chromatogr A. Elsevier B.V.; 2011;1218: 3771–3780.
21. De Souza PP, Cardeal ZDL, Augusti R, Morrison P, Marriott PJ. Determination of volatile compounds in Brazilian distilled cachaça by using comprehensive two-dimensional gas chromatography and effects of production pathways. J Chromatogr A. 2009;1216: 2881–2890.
22. Kallio M, Jussila M, Rissanen T, Anttila P, Hartonen K, Reissell A, et al. Comprehensive two-dimensional gas chromatography coupled to time-of-flight mass spectrometry in the identification of organic compounds in atmospheric aerosols from coniferous forest. J Chromatogr A. 2006;1125: 234–243.
23. Zehentbauer G, Reineccius GA. Determination of key aroma components of Cheddar cheese using dynamic headspace dilution assay. Flavour Fragr J. 2002;17: 300–305.
24. Beal AD, Mottram DS. Compounds contributing to the characteristic aroma of malted barley. J Agric Food Chem. American Chemical Society; 1994;42: 2880–2884.
25. Methven L, Tsoukka M, Oruna-Concha MJ, Parker JK, Mottram DS. Influence of sulfur amino acids on the volatile and nonvolatile components of cooked salmon (Salmo salar). J Agric Food Chem. 2007;55: 1427–1436.
26. Borse BB, Rao LJM, Ramalakshmi K, Raghavan B. Chemical composition of volatiles from coconut sap (neera) and effect of processing. Food Chem. 2006;101: 877–880.
27. Xu X, van Stee LLP, Williams J, Beens J, Adahchour M, Vreuls RJJ, et al. Comprehensive two-dimensional gas chromatography (GCxGC) measurements of volatile organic compounds in the atmosphere. Atmos Chem Phys Discuss. 2003;3: 1139–1181.
28. Tellez MR, Canel C, Rimando AM, Duke SO. Differential accumulation of isoprenoids in glanded and glandless Artemisia annua L. Phytochemistry. 1999;52: 1035–1040.

29. Solina M, Baumgartner P, Johnson RL, Whitfield FB. Volatile aroma components of soy protein isolate and acid-hydrolysed vegetable protein. Food Chem. 2005;90: 861–873.
30. Ceccarini L, Macchia M, Flamini G, Cioni PL, Caponi C, Morelli I. Essential oil composition of Helianthus annuus L. leaves and heads of two cultivated hybrids “Carlos” and “Florom 350.” Ind Crops Prod. 2004;19: 13–17.
31. Ferhat MA, Tigrine-Kordjani N, Chemat S, Meklati BY, Chemat F. Rapid Extraction of Volatile Compounds Using a New Simultaneous Microwave Distillation: Solvent Extraction Device. Chromatographia. 2007;65: 217–222.
32. Pino JA, Marbot R, Fuentes V. Characterization of volatiles in bullock’s heart (Annona reticulata L.) fruit cultivars from Cuba. J Agric Food Chem. 2003;51: 3836–3839.
33. Mevy JP, Bessiere JM, Pelissier Y, Masotti V, Ruzzier M, Rabier J, et al. Composition of the volatile constituents of the aerial parts of an endemic plant of Ivory Coast, Monanthotaxis capea (E.G. & A. Campus) verdc. Flavour Fragr J. 2004;19: 526–528.
34. Shang C, Yaoming H, Deng C, Hu K. Rapid determination of volatile constituents of Michelia alba flowers by gas chromatography-mass spectrometry with solid-phase microextraction. J Chromatogr A. 2002;942: 283–288.
35. Petronilho S, Rocha SM, Ramírez-chávez E, Molina-torres J, Rios-chavez P. Assessment of the terpenic profile of Callistemon citrinus (Curtis) Skeels from Mexico. Ind Crop Prod. Elsevier B.V.; 2013;46: 369–379.
36. Rocha SM, Coutinho P, Delgadillo I, Coimbra MA. Headspace-solid phase microextraction-gas chromatography as a tool to define an index that establishes the retention capacity of the wine polymeric fraction towards ethyl esters. J Chromatogr A. 2007;1150: 155–161.
37. Rocha SM, Coelho E, Zrostlíková J, Delgadillo I, Coimbra MA. Comprehensive two-dimensional gas chromatography with time-of-flight mass spectrometry of monoterpenoids as a powerful tool for grape origin traceability. J Chromatogr A. 2007;1161: 292–9.
38. Jalali HT, Petronilho S, Villaverde JJ, Coimbra MA, Domingues MRM, Ebrahimian ZJ, et al. Deeper insight into the monoterpenic composition of Ferula gummosa oleo-gum-resin from Iran. Ind Crops Prod. Elsevier B.V.; 2012;36: 500–507.
39. Doimo L. Iso-Citrals and Iso-Geraniols in Lemon-Myrtle (Backhousia citriodora F. Muell.) Essencial Oils. J Essent Oil Res. 2001;13: 236–237.
40. Beaulieu JC, Grimm CC. Identification of volatile compounds in cantaloupe at various developmental stages using solid phase microextraction. J Agric Food Chem. 2001;49: 1345–1352.
41. Ozel MZ, Gogus F, Lewis AC. Comparison of direct thermal desorption with water distillation and superheated water extraction for the analysis of volatile components of Rosa damascena Mill. using GCxGC-TOF/MS. Anal Chim Acta. 2006;566: 172–177.
42. Petronilho S, Maraschin M, Delgadillo I, Coimbra MA, Rocha SM. Sesquiterpenic composition of the inflorescences of Brazilian chamomile (Matricaria recutita L.): Impact of the agricultural practices. Ind Crops Prod. 2011;34: 1482–1490.
43. Jalali HT, Petronilho S, Villaverde JJ, Coimbra MA, Domingues MRM, Ebrahimian ZJ, et al. Assessment of the sesquiterpenic profile of Ferula gummosa oleo-gum-resin (galbanum) from Iran. Contributes to its valuation as a potential source of sesquiterpenic compounds. Ind Crops Prod. Elsevier B.V.; 2013;44: 185–191.
44. El-Sayed AM, Heppelthwaite VJ, Manning LM, Gibb AR, Suckling DM. Volatile constituents of fermented sugar baits and their attraction to lepidopteran species. J Agric Food Chem. 2005;53: 953–958.
45. Steinhaus P, Schieberle P. Characterization of the key aroma compounds in soy sauce using approaches of molecular sensory science. J Agric Food Chem. 2007;55: 6262–6269.
46. Isidorov VA, Krajewska U, Dubis EN, Jdanova MA. Partition coefficients of alkyl aromatic hydrocarbons and esters in a hexane-acetonitrile system. J Chromatogr A. 2001;923: 127–136.
47. Bicalho B, Pereira AS, Aquino Neto FR, Pinto AC, Rezende CM. Application of high-temperature gas chromatography-mass spectrometry to the investigation of glycosidically bound components related to cashew apple (Anacardium occidentale L. var. nanum) Volatiles. J Agric Food Chem. 2000;48: 1167–1174.
48. Valim MF, Rouseff RL, Lin J. Gas Chromatographic − Olfactometric Characterization of Aroma Compounds in Two Types of Cashew Apple Nectar Gas Chromatographic − Olfactometric Characterization of Aroma Compounds in Two Types of Cashew Apple Nectar. J Agric Food Chem. 2003;51: 1010–1015.
49. Yuping Z, Jiming LI, Yan XU, Hui D, Wenlai FAN, Guang Z. Extraction , preparation and identification of volatile compounds in Changyu XO Brandy. Chinese J Chromatogr. 2008;26: 212–222.
50. Sotomayor JA, Martínez R, García A., Jordán MJ. Thymus zygis Subsp . Gracilis: Watering Level Effect on Phytomass Production and Essential Oil Quality. J Agric Food Chem. 2004;52: 5418–5424.
51. Olson KL, Wong CA, Fleck LL, Lazar DF. Qualitative and Quantitative Determination of Solvent Formulations in Automotive Paints. J Chromatogr Sci. 1987;25: 418–423.
52. Vasta V, Ratel J, Engel E. Mass Spectrometry Analysis of Volatile Compounds in Raw Meat for the Authentication of the Feeding Background of Farm Animals. J Agric Food Chem. 2007;55: 4630–4639.
53. Choi H-S. Character impact odorants of Citrus Hallabong [(C. unshiu Marcov ×C. sinensis Osbeck) xC. reticulata Blanco] cold-pressed peel oil. J Agric Food Chem. 2003;51: 2687–2692.
54. Demyttenaere JCR, Martínez JIS, Verhé R, Sandra P, De Kimpe N. Analysis of volatiles of malt whisky by solid-phase microextraction and stir bar sorptive extraction. J Chromatogr A. 2003;985: 221–232.
55. Marques F de A, McElfresh JS, Millar JG. Kováts Retention Indexes of Monounsaturated C12, C14, and C16 Alcohols, Acetates and Aldehydes Commonly Found in Lepidopteran Pheromone Blends. J Braz Chem Soc. 2000;11: 592–599.
56. Guichard H, Lemesle S, Ledauphin J, Barillier D, Picoche B. Chemical and sensorial aroma characterization of freshly distilled calvados. 1. Evaluation of quality and defects on the basis of key odorants by olfactometry and sensory analysis. J Agric Food Chem. 2003;51: 424–432.
57. Fan W, Qian MC. Characterization of aroma compounds of Chinese “Wuliangye” and “Jiannanchun” liquors by aroma extract dilution analysis. J Agric Food Chem. 2006;54: 2695–2704.
58. Verevkin SP, Krasnykh EL, Vasiltsova T V, Heintz A. Determination of Ambient Temperature Vapor Pressures and Vaporization Enthalpies of Branched Ethers. J Chem Eng Data. 2003;48: 591–599.
59. Santos MC, Nunes C, Rocha MAM, Rodrigues A, Rocha SM, Saraiva JA, et al. Impact of high pressure treatments on the physicochemical properties of a sulphur dioxide-free white wine during bottle storage: Evidence for Maillard reaction acceleration. Innov Food Sci Emerg Technol. Elsevier Ltd; 2013;20: 51–58.
60. Hofmann T, Schieberle P. Identification of key aroma compounds generated from cysteine and carbohydrates under roasting conditions. Zeitschrift für Leb und -forsch A. 1998;207: 229–236.
61. Zaikin VG, Borisov RS. Chromatographic – Mass Spectrometric Analysis of Fischer – Tropsch Synthesis Products. J Anal Chem. 2002;57: 544–551.
62. Rocha SM, Caldeira M, Carrola J, Santos M, Cruz N, Duarte IF. Exploring the human urine metabolomic potentialities by comprehensive two-dimensional gas chromatography coupled to time of flight mass spectrometry. J Chromatogr A. Elsevier B.V.; 2012;1252: 155–63.
63. Buchin S, Salmon J-C, Carnat A-P, Berger T, Bugaud C, Bosset JO. Identification de composés monoterpéniques, sesquiterpéniques et benzéniques dans un lait d’alpage très riche en ces substances. Mitt Leb Hyg. 2002;93: 199–216.

64. Adams RP. Systematics of Juniperus section Juniperus based on leaf essential oils and random amplified polymorphic DNAs (RAPDs). Biochem Syst Ecol. 2000;28: 515–528.
65. Angioni A, Barra A, Coroneo V, Dessi S, Cabras P. Chemical composition, seasonal variability, and antifungal activity of Lavandula stoechas L. ssp. stoechas essential oils from stem/leaves and flowers. J Agric Food Chem. 54AD;12: 4364–4370.
66. Boylston TD, Viniyard BT. Isolation of volatile flavor compounds from peanut butter using purge-and-trap technique. In: Wetzel D, Charalambous G, editors. Instrumental Methods in Food and Beverage Analysis. 1998. pp. 225–243.
67. Gallois A, Grimont PAD. Pyrazines responsible for the potatolike odor produced by some Serratia and Cedecea strains. Appl Environ Microbiol. 1985;50: 1048–1051.
68. Murkovic M, Piironen V, Lampi AM, Kraushofer T, Sontag G. Changes in chemical composition of pumpkin seeds during the roasting process for production of pumpkin seed oil (Part 1: Non-volatile compounds). Food Chem. 2004;84: 359–365.
69. Oruna-Concha, M.J. Duckham SC, Ames JM. Comparison of volatile compounds isolated from the skin and flesh of four potato cultivars after baking. J Agric Food Chem. 2001;49: 2414–2421.
70. Qian M, Reineccius G. Potent aroma compounds in Parmigiano Reggiano cheese studied using a dynamic headspace (purge-trap) method. Flavour Fragr J. 2003;18: 252–259.
71. Fan W, Xu Y, Zhang Y. Characterization of pyrazines in some Chinese liquors and their approximate concentrations. J Agric Food Chem. 2007;55: 9956–9962.
72. Engel E, Baty C, Le Corre D, Souchon I, Martin N. Flavor-active compounds potentially implicated in cooked cauliflower acceptance. J Agric Food Chem. 2002;50: 6459–6467.
73. Andersson JT, Weis U. Gas chromatographic determination of polycyclic aromatic compounds with fluorinated analogues as internal standards. J Chromatogr A. 1994;659: 151–161.
